# Novel regulation mechanism of adrenal cortisol and DHEA biosynthesis via the endogen ERAD inhibitor small VCP-interacting protein

**DOI:** 10.1038/s41598-022-04821-y

**Published:** 2022-01-18

**Authors:** Recep Ilhan, Göklem Üner, Sinem Yilmaz, Esra Atalay Sahar, Sevil Cayli, Yalcin Erzurumlu, Oguz Gozen, Petek Ballar Kirmizibayrak

**Affiliations:** 1 Department of Biochemistry, Faculty of Pharmacy, Ege University, 35100 Bornova, Izmir Turkey; 2 Department of Bioengineering, Izmir Institute of Technology, 35430 Urla, Izmir Turkey; 3 Department of Biotechnology, Graduate School of Natural and Applied Sciences, Ege University, Izmir, Turkey; 4 Department of Bioengineering, Faculty of Engineering, University of Alanya Aladdin Keykubat, Antalya, Turkey; 5 Department of Histology and Embryology, Medical Faculty, Ankara Yıldırım Beyazıt University, Ankara, Turkey; 6 Department of Physiology, School of Medicine, Ege University, Izmir, Turkey; 7 Present Address: Suleyman Demirel University, Faculty of Pharmacy, Isparta, Turkey

**Keywords:** ER-associated degradation, Adrenal cortex hormones

## Abstract

Endoplasmic reticulum-associated degradation (ERAD) is a well-characterized mechanism of protein quality control by removal of misfolded or unfolded proteins. The tight regulation of ERAD is critical for protein homeostasis as well as lipid metabolism. Although the mechanism is complex, all ERAD branches converge on p97/VCP, a key protein in the retrotranslocation step. The multifunctionality of p97/VCP relies on its multiple binding partners, one of which is the endogenous ERAD inhibitor, SVIP (small VCP-interacting protein). As SVIP is a promising target for the regulation of ERAD, we aimed to assess its novel physiological roles. We revealed that SVIP is highly expressed in the rat adrenal gland, especially in the cortex region, at a consistently high level during postnatal development, unlike the gradual increase in expression seen in developing nerves. Steroidogenic stimulators caused a decrease in SVIP mRNA expression and increase in SVIP protein degradation in human adrenocortical H295R cells. Interestingly, silencing of SVIP diminished cortisol secretion along with downregulation of steroidogenic enzymes and proteins involved in cholesterol uptake and cholesterol biosynthesis. A certain degree of SVIP overexpression mainly increased the biosynthesis of cortisol as well as DHEA by enhancing the expression of key steroidogenic proteins, whereas exaggerated overexpression led to apoptosis, phosphorylation of eIF2α, and diminished adrenal steroid hormone biosynthesis. In conclusion, SVIP is a novel regulator of adrenal cortisol and DHEA biosynthesis, suggesting that alterations in SVIP expression levels may be involved in the deregulation of steroidogenic stimulator signaling and abnormal adrenal hormone secretion.

## Introduction

p97/VCP (CDC48p in yeast) is a highly abundant molecular chaperone that functions in diverse cellular processes such as ubiquitin-dependent proteolysis, retrograde transport of protein from the endoplasmic reticulum (ER), cell cycle progression, DNA replication, chromosome condensation, and autophagy^1–3^. The functional versatility of p97/VCP is mainly due to the presence of its multiple interacting proteins. For instance, while the p47-p97/VCP complex functions in Golgi assembly during mitosis, the Ufd1-Npl4-p97/VCP complex participates in endoplasmic reticulum-associated degradation (ERAD), a process that not only functions as a quality control mechanism by removing unfolded proteins from the ER, but also regulates the abundance of properly folded proteins^4^. The interaction of p97/VCP with the ubiquitin ligase gp78 and the putative retrotranslocation channel Derlin1 is essential for the degradation of ERAD substrates^2,5^. SVIP (small p97/VCP-interacting protein) is a membrane-anchored 76-amino acid protein that binds to p97/VCP in a mutually exclusive manner of p47, Ufd1-Npl4, and gp78^4,6^. Both SVIP and gp78 directly interact with p97/VCP via their shared p97/VCP-interacting motif. SVIP was identified as the first endogenous ERAD inhibitor through its regulatory role in the formation of the ERAD machinery that includes p97/VCP, gp78, and Derlin1^6^. Overexpression of SVIP inhibits gp78-mediated ERAD by uncoupling gp78 from its functional partners, namely p97/VCP and Derlin-1, therefore suppressing the degradation of gp78’s ERAD substrates CD3δ, the Z variant of α-1-antitrypsin, and CFTRΔF508^6,7^. Interestingly, prolonged ER stress significantly upregulates SVIP, which inhibits ERAD and may promote autophagy^8^. Indeed, it has been shown that SVIP regulates the autophagy process by modulating LC3 processing, p62 expression, and sequestration of polyubiquitinated proteins into autophagosomes^8^.

Mounting evidence indicates that similar to p97/VCP, SVIP is also a multifunctional protein. SVIP regulates the size of lipid droplets in fibrotic rat liver by modulating the levels of Rab7, a protein that plays critical role in the fusion of lysosomes with autophagosomes as well as lipid droplets^9^. Concomitantly, SVIP was reported to be present in the proteome of VLDL transport vesicles, where it colocalizes and interacts with apolipoprotein-B100^10^. Furthermore, silencing of SVIP in hepatocytes caused a significant decrease in VLDL transport vesicle formation and VLDL secretion^11^. Besides its regulatory role in the lipid metabolism of hepatocytes, SVIP was shown to be highly expressed in the cerebrum and cerebellum of the brain in a tissue distribution assay using tissue extracts made from different mouse organs^8^. Intriguingly, SVIP expression was highly correlated with levels of myelin basic protein in the developing nerves, while it was barely detectable in the early postnatal stage and strongly increased at later stages^12^. Moreover, while SVIP and p97/VCP were co-localized in neuronal cell bodies, they are not co-localized in the peripheral nerve myelin, suggesting that SVIP localizes and functions in compact myelin in a manner independent of its interactions with p97/VCP^12^.

There are several additional studies by our group and others highlighting the possible role of SVIP in tumorigenesis as well. Firstly, the ERAD pathway is reported to be regulated by androgen in androgen-responsive prostate cancer cells^13^. Regulation of the levels of ERAD components leads to enhanced ERAD proteolytic activity, which was found to be positively related with prostate tumorigenesis^13^. Moreover, this androgen-mediated downregulation of SVIP was also reported to be present in the glioma cells and involved in the cell proliferation regulation of glioma cells with wild-type p53^14^. Lastly, proteomics and metabolomics analysis revealed that epigenetic loss of SVIP induces metabolic programming of cancer cells via depletion of mitochondrial enzymes and oxidative respiration activity^15^.

Herein, we report for the first time that SVIP is highly expressed in the rat adrenal gland tissue and is involved in the regulation of human adrenal cortisol and dehydroepiandrosterone (DHEA) biosynthesis. We investigated the postnatal developmental levels and zonal expression pattern of SVIP using rat adrenal gland tissues, while the functional role of SVIP in adrenal gland was studied in the human adrenal corticocarcinoma H295R cell line, which is generally used to study the adrenal cortex biology in vitro. Our findings indicate a novel role for SVIP in cortisol and DHEA biosynthesis and the homeostasis of adrenal cortex cells.

## Material and methods

### Materials

The H295R cell line was grown in DMEM-F12 medium (GIBCO) with Nu serum and ITS-Premix (Corning). Antibodies against SVIP (Sigma-Aldrich, HPA039807), CYP17A1 (Santa Cruz, sc-374244), CYP11A1(CST, 14217), CYP11B1 (ABCAM, Ab-229884), HSD3β2 (ABCAM, Ab-75710), HMGR (ABCAM, Ab-174830), StAR (ABCAM, Ab-58013), LDLR (ABCAM, Ab-52818), caspase 3 (CST, 9665), cleaved-caspase 3 (CST, 9664), PARP-1 (CST, 9542), eIF2α (CST, 9722), p-eIF2α (CST, 9721), anti-LC3 (CST, 12741), anti-p62 (CST, 5114), actin (Sigma-Aldrich, A5316), and GAPDH (CST, 5174) were used for protein quantity determination by immunoblotting. The Organelle Localization IF Antibody Sampler Kit (CST, 8653) was used in immunofluorescence co-localization experiments. HRP-conjugated anti-mouse or anti-rabbit IgG was purchased from Pierce.

Cycloheximide (66-81-9) was purchased from Calbiochem and 8Br-cAMP (Ab-141448), forskolin (Ab-120058), and angiotensin II (Ab-120183) were purchased from ABCAM.

### Immunohistochemistry

Laboratory animals were obtained from the Gaziosmanpasa University Experimental Animal Research Laboratory and the experimental procedures were reviewed and approved by the Gaziosmanpasa University ethics committee (No: 2014 HADYEK-004). The study was conducted in accordance with the National Research Council’s Guide for the Care and Use of Laboratory Animals and the ARRIVE guidelines 2.0. Same-age animals were euthanized by administering an overdose of sodium pentobarbital (150 mg/kg, intraperitoneally [ip]) before organ removal. One adrenal gland from each animal was fixed in 4% formalin for 12 h immediately upon collection, then dehydrated and embedded in paraffin for immunohistochemistry. The contralateral adrenal gland from each animal was frozen for protein analysis.

Immunohistochemistry was performed according to a previously described procedure^16^. Briefly, the serial sections, 5 µm thick, were collected on poly-l-lysine-coated slides (Sigma-Aldrich) and incubated overnight at 56 °C. The tissue sections were then deparaffined with xylene and then rehydrated with ethanol. The sections were treated with 10 mmol/L citrate buffer (pH 6.0) twice for 5 min in the microwave oven and allowed to cool for 20 min. Endogenous peroxidase activity was blocked using 3% hydrogen peroxide for 20 min. The tissue section slides were incubated with polyclonal anti-SVIP antibody (1:100) at room temperature for 1 h. Negative control sections were treated with an isotype of mouse IgG and rabbit IgG antibodies. Next, the samples were incubated with HRP-conjugated secondary antibody (BA-1000; 1:400; Vector Laboratories) and DAB chromogen (Sigma, Laboratories, Utah) added sequentially. The sections were stained simultaneously with Mayer hematoxylin (ScyTek Laboratories) and microscope images were obtained with a Leica microscope (Leica DM2500, Nussloch, Germany).

### Cell culture and treatments

Human adrenocortical carcinoma cell line H295R was obtained from American Type Culture Collection (ATCC, USA). Routine growth and experimental procedures with the H295R cell line were carried out in accordance with ATCC and OECD standard operating procedures. Briefly, the cells were cultured in 100-mm cell culture dishes in Dulbecco’s modified Eagle medium/HamF12 cell culture medium supplemented with 1% ITS Premix and 2.5% Nu Serum. The medium was changed once every 2–3 days. The cells were passaged when the cell density reached 80% and used in experiments after 5 to 15 passages.

All chemicals used, including steroidogenic stimulating agents, were prepared at 1000× concentration so that the DMSO ratio did not exceed 0.1%.

The Lipofectamine 3000 kit (Invitrogen) was used following the manufacturer’s instructions, in order to manipulate protein expression levels either by overexpression or silencing. Silencer^®^ Negative Control siRNA (Ambion, 4611) and SVIP siRNA (AM16104, sense sequence: GACAAAAAGAGGCUGCAUC) were ordered from Ambion. The pCIneo-SVIP-His plasmid has been previously reported^6^.

For the cycloheximide experiment, cells transfected with SVIP-encoding plasmid or treated with forskolin were incubated with 100 µg/ml cycloheximide (Calbiochem) for the indicated time periods. At the end of the experiment, the cells were harvested and the proteins of interest were examined by immunoblotting.

### Double immunofluorescence

H295R cells or cryostat sections of rat adrenal cortex were fixed with 4% paraformaldehyde in 1× PBS at 4 °C for 30 min, and then subjected to immunofluorescent staining as previously reported^2^.

### Preparation of protein samples and immunoblotting

Cell lysates were prepared using RIPA buffer (1X PBS, 1% nonidet P-40, 0.5% sodium deoxycholate, and 0.1% SDS, pH 8.0). The total protein content was determined using bicinchoninic acid (BCA) protein assay (Thermo Fisher Scientific, USA). Samples (typically 40 µg) were loaded onto gels after 1-h treatment with 4× Laemmli buffer at 37 °C. Following SDS-PAGE, gels were transferred to the PVDF membrane, which was then, PVDF membrane was treated with primary antibodies and secondary antibodies to detect the protein of interest. Proteins were visualized with a Vilber Loumart FX-7 (Vilber Lourmat, Thermo Fisher Scientific, US) using the chemiluminescence method for protein quantification and analyzed with ImageJ software (http://imagej.nih.gov/ij/).

### Total RNA isolation and RT-PCR experiments

Biorad Aurum Total RNA Mini Kit (Bio-Rad, USA) was used for RNA extraction as per the manufacturer's instructions. Prior to cDNA synthesis, RNA concentrations were determined on a Beckman Coulter Du730 instrument capable of measuring at 260/280 nm wavelength. The iScript cDNA Synthesis Kit (Bio-Rad, USA) was used to obtain cDNA from 1 µg of RNA according to the instructions. Quantitative RT-PCR was performed using SYBR Green I (Bio-Rad, USA) and LightCycler480 thermocycler (Roche). Gene expression analysis was conducted using specific primers for CYP11A1 (F: GCTTTGCCTTTGAGTCCATCA, R: CTCGGGGTTCACTACTTCCTC), CYP11B1 (F: GGACCCACCTCTTGTTTCATAG, R: GAATGGAACTGGCGTCCTTAT), CYP17A1 (F: TCACAATGAGAAGGAGTGGCAC, R: TACTGACGGTGAGATGAGCTGG), HSD3β2 (F: CAGAGATGTGCATGTGGGTAT, R: GTTGGGCATTGTGTGAAAGAG), LDLR (F: GCCTCTGAAATGCCTCTTCT, R: CCCAGAAGCCACTCATACATAC), HMGR (F: TGATTGACCTTTCCAGAGCAAG, R: CTAAAATTGCCATTCCACGAGC), CYP21A2 (F: CAAGCTGGTGTCTAGGAACTACC, R: TCTCATGCGCTCACAGAACTC), and SVIP (F: CAAAAAGAGGCTGCATCTCGG, R: AACTGTCCACCTAAGTCCACC) in 10-μL reactions with 300 nM of primer pairs. Fold change for the transcripts was normalized against the housekeeping genes; TBP and 36B4. The Ct values (threshold cycle value) determined for each sample were analyzed with the QCt relative quantification method using the Qiagen REST 2009 program. At least two independent biological replicates with three technical replicates per experiment were used for each PCR.

### Flow cytometry

Cell flow cytometry experiments were conducted using the PE Annexin V Apoptosis Detection Kit I (BD Biosciences, 559763) according to the manufacturer’s instructions. Briefly, transfected cells were suspended in 100 μl of Annexin V binding buffer and then 5 μl of FITC-conjugated Annexin V and 7-AAD added to samples. After the incubation at room temperature for 15 min in the dark, the stained cells were examined using a FacsCanto Flow Cytometry (BD Bioscience, USA).

### Hormone analysis

As stated in the OECD document and various literature, hormone analyses were performed using H295R cells with 4 to 10 passages. Cells were grown on 6-well plates and transfected as indicated. The media was replenished 6 or 24 h post-transfection of plasmid encoding SVIP or SVIP siRNA, respectively. The growth media and cell lysates were collected 48 h later. Cortisol and DHEA levels were determined using ELISA kits (Enzo Life Sciences, ADI-900-071 and ADI-900-093), and hormone concentrations were normalized to the total protein concentration and calculated as a fold change^17,18^.

### Statistical analysis

Data are presented as means ± standard deviation (SD). One-way ANOVA with post hoc test or Student’s t-test was performed for statistical analyses by using GraphPad Prism software. The significance threshold was accepted as p < 0.05.

## Results

### SVIP is highly expressed in adrenal gland

**Figure 1:**
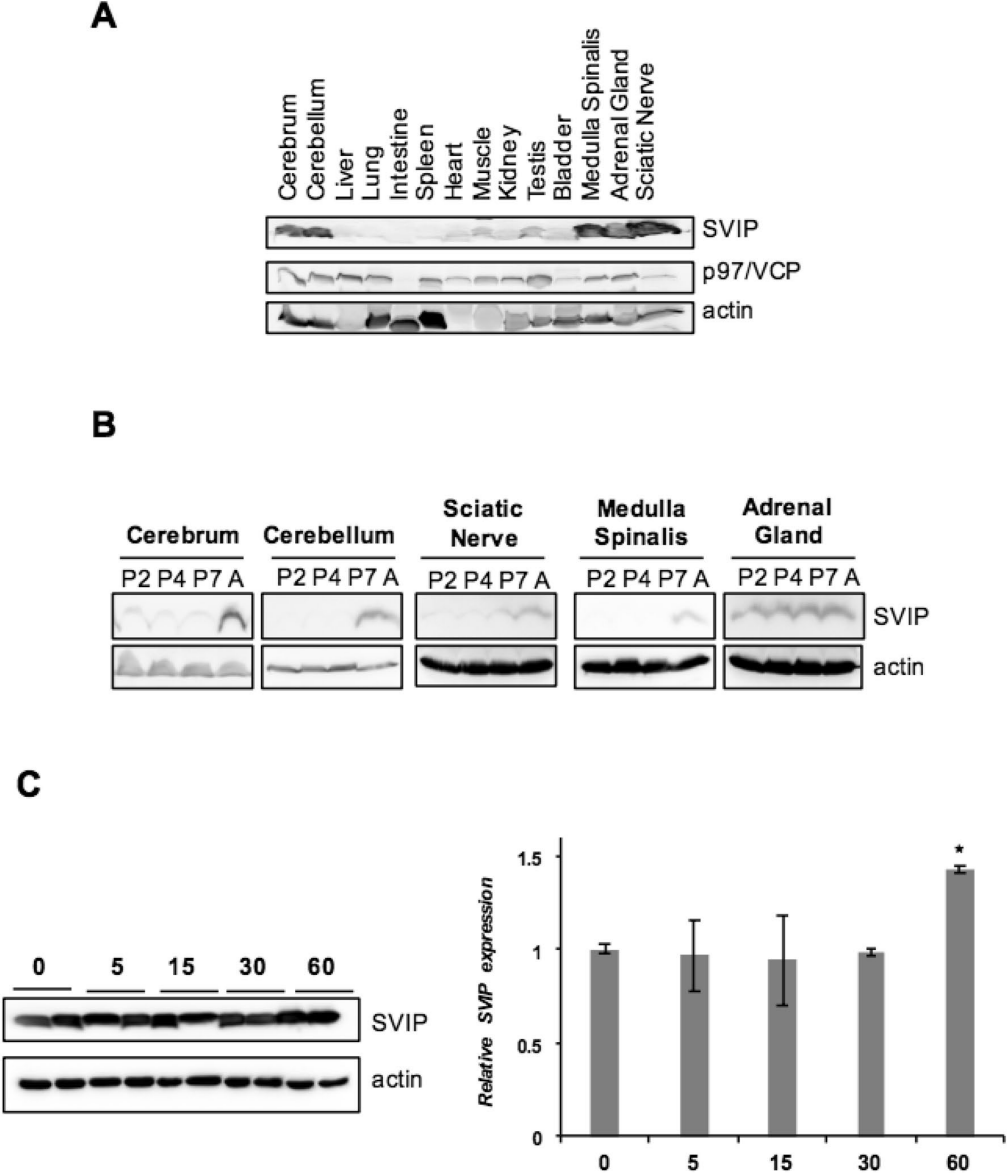
SVIP is highly expressed in adrenal gland. (**A**) Equal amounts of total protein from each organ extract were analyzed by immunoblotting (IB) with antibodies against SVIP and p97/VCP. (**B**) Mouse tissues were obtained at postnatal day 2, 4, 7, and 28 and processed for IB. (**C**) SVIP expression was investigated in the adrenal glands of rats at the indicated ages. The left shows representative Western blot images and the right shows the results of quantitative densitometric analysis of the changes in SVIP protein expression. Error bars indicate standard deviation (*p < 0.05).

We first examined the tissue distribution of SVIP using 14 different mouse tissue extracts. Our results revealed that SVIP is highly expressed in the medulla spinalis and adrenal gland in addition to the previously reported cerebrum, cerebellum, and sciatic nerve^8^ (Fig. 1A). The expression of SVIP was particularly high in adult tissues but very low at postnatal day 2, 4, and 7 in the medulla spinalis as well as the cerebrum, cerebellum, and sciatic nerve as previously reported, suggesting that SVIP may function in one or many of the changes that occur during postnatal maturation of the central nervous system. Interestingly, SVIP was expressed at a low level in a subset of other non-neuronal adult tissues other than the adrenal gland. Unlike in the central nervous system, SVIP was highly expressed in the adrenal gland in all postnatal development stages (Fig. 1B). A similar developmental expression pattern was observed in rat adrenal gland, with a slight increase at day 60 (Fig. 1C).

**Figure 2:**
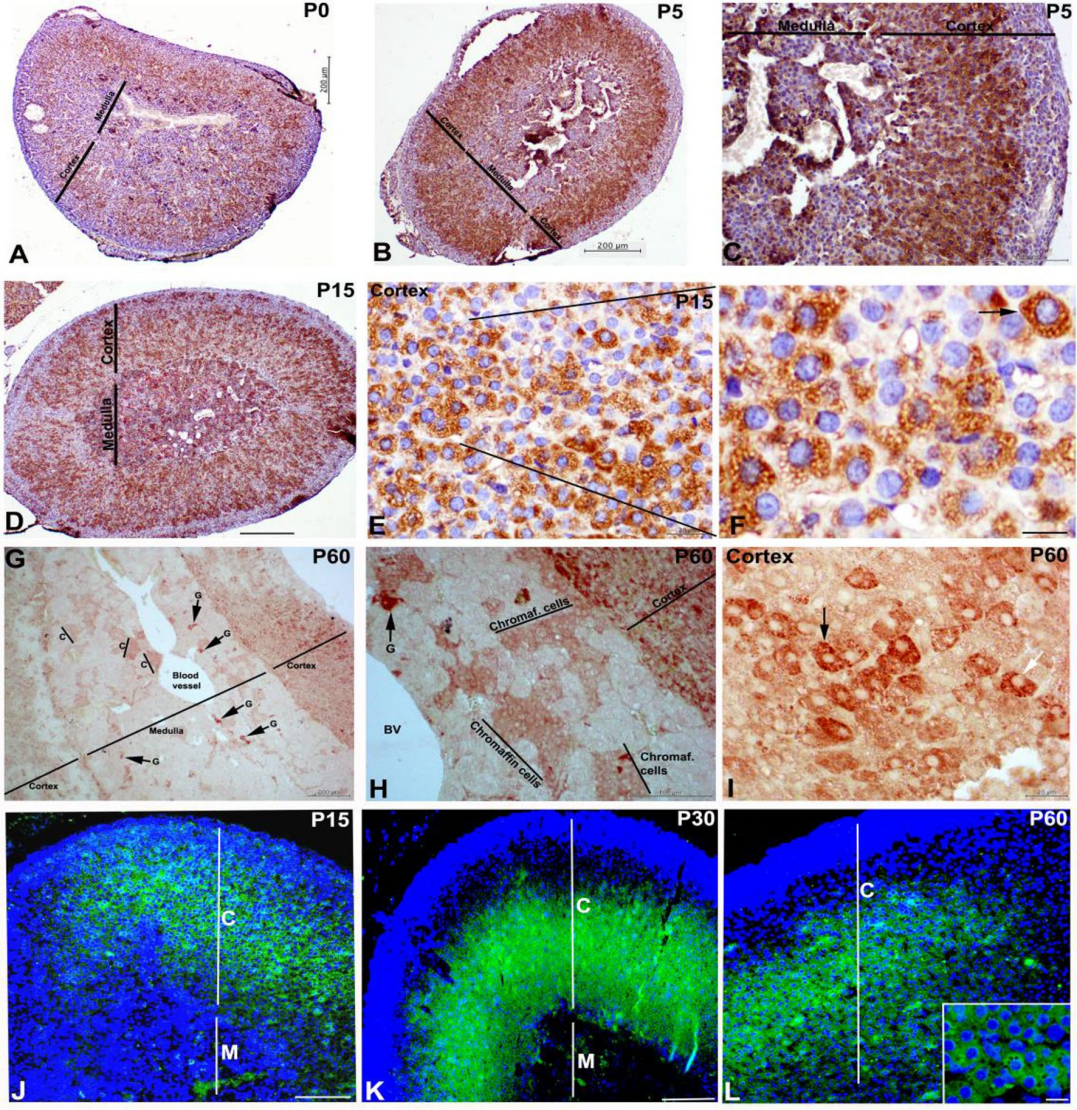
Distribution of SVIP in the rat adrenal gland during the postnatal development. SVIP immunoreactivity was detected in the rat adrenal gland at postnatal day 0 (**A**), 5 (**B**,**C**), 15 (**D**–**F**), and 60 (**G**–**I**) by IHC. Strong expression of SVIP is observed in the cortex (**C**) compared to medulla (**M**) in postnatal rat adrenal glands. SVIP immunopositivity is seen in the secretory vesicles (arrows) of adrenal cortex cells (arrows, **F** and **I**). SVIP localization was also determined by immunofluorescence labeling in the rat adrenal gland at postnatal day 15 (**J**), 30 (**K**), and 60 (**L**). Scale bars: 200 µm (**A**,**B**,**D**,**G**,**J**,**K**); 100 µm (**C**,**H**); 20 µm (**E**,**F**,**I**,**L**).

Next, we determined the localization of SVIP in rat adrenal gland by immunohistochemistry (IHC). At postnatal day 0, SVIP immunoreactivity was strongly identified in the inner zones but not in the outer layer of the adrenal cortex, i.e. the zona glomerulosa (Fig. 2A). Weak SVIP immunoreactivity was observed in the adrenal medulla. The strong expression pattern of SVIP in the cortex persisted at postnatal day 5 and 15 (Fig. 2B–F). At 60 days of postnatal age, SVIP expression was dominant in the cortex of the adrenal gland but weak expression in the medulla of the adrenal gland (Fig. 2G–I). SVIP expression was found to be specific to the neuroendocrine chromaffin cells of the adrenal medulla by day 60 (Fig. 2G,H). In line with IHC data, immunofluorescence labeling of SVIP also showed that SVIP was mainly expressed in the inner zones of the rat adrenal cortex but not in the zona glomerulosa (Fig. 2J,K). Together, these data indicate that SVIP is preferentially expressed in the zona fasciculata/reticularis (ZF/R) of the rat adrenal gland.

**Figure 3:**
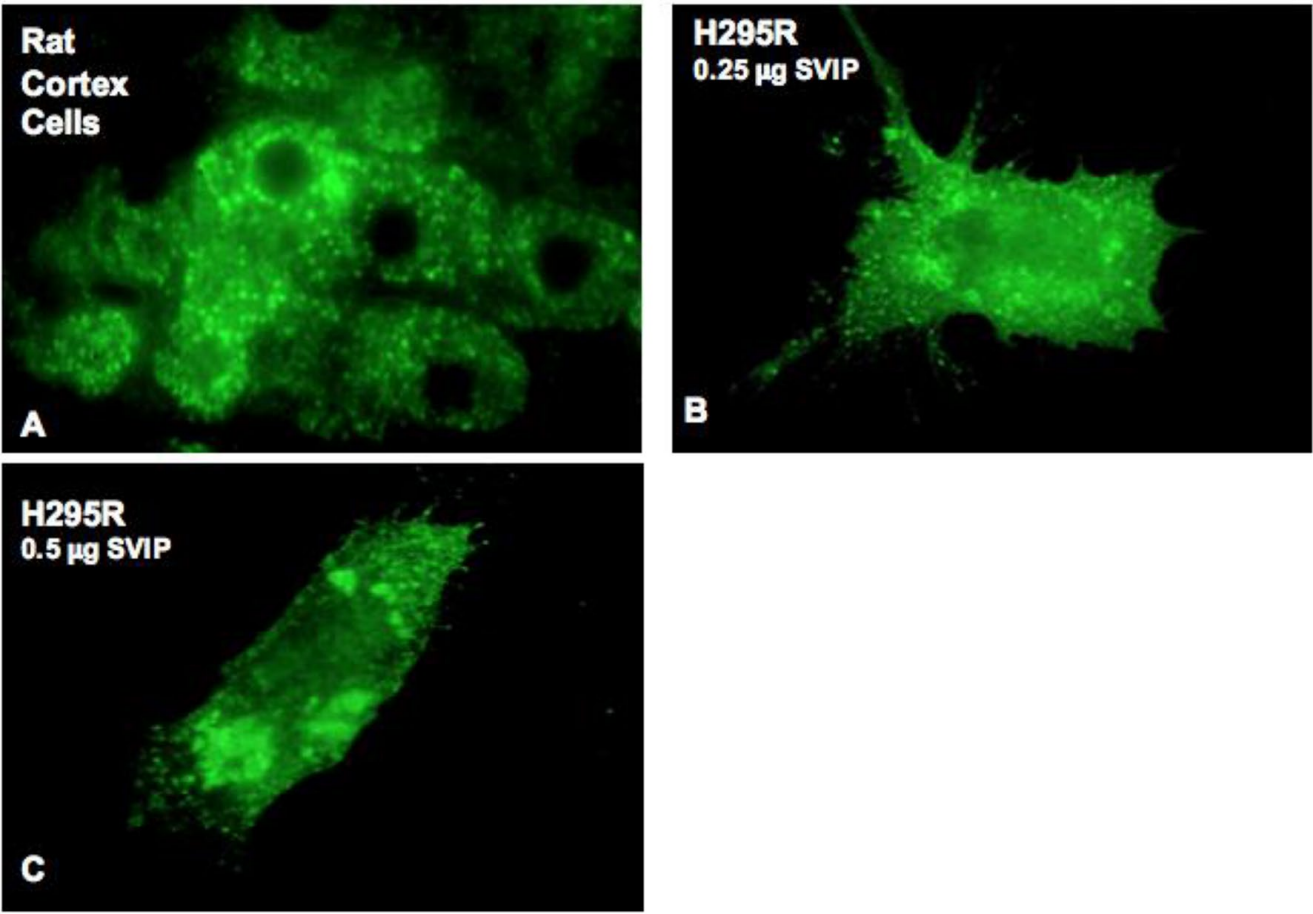
SVIP is localized at punctate structures as juxtanuclear vacuoles in rat adrenal cortex cells and H295R cell line. (**A**) Location of endogenous SVIP was determined in postnatal day 60 rat adrenal cortex cells by immunostaining using confocal microscopy. (**B**,**C**) H295R cells were transfected with (**B**) 0.25 μg or (**C**) 0.5 μg pCIneo-SVIP-His plasmid, then cells were stained with anti-His antibody.

After the initial SVIP expression screening in rodent organs and the detection of adrenal cortex as the only tissue that expresses SVIP at all developmental stages, we aimed to analyze the role of SVIP in adrenal function. However, there are significant differences between rodent and human adrenal physiology. Both the zonation and the repertoire of steroidogenic enzymes vary between rodents and human^19,20^. Since the adrenal cortex of adult mice and rats lacks CYP17A1, corticosterone is the principal glucocorticoid secreted, and adrenal androgens are not biosynthesized in rodents. Thus, to further study the role and regulation of SVIP in adrenal cortex cells, H295R human pluripotent adrenocortical cells were used as an in vitro model because they express all the steroidogenic enzymes and have steroid hormone secretion and regulation pattern mimicking primary cultures of adrenal cortex cells^21^. Immunofluorescence analysis revealed that SVIP was localized in numerous punctuated structures on P60 rat adrenal cortex cells (Fig. 3A), and SVIP was also associated with various small and large punctate structures in H295R cells (Fig. 3B,C). Furthermore, SVIP did not co-localize with CYP17A1 in H295R cells (Supplementary Fig. 1).

**Figure 4:**
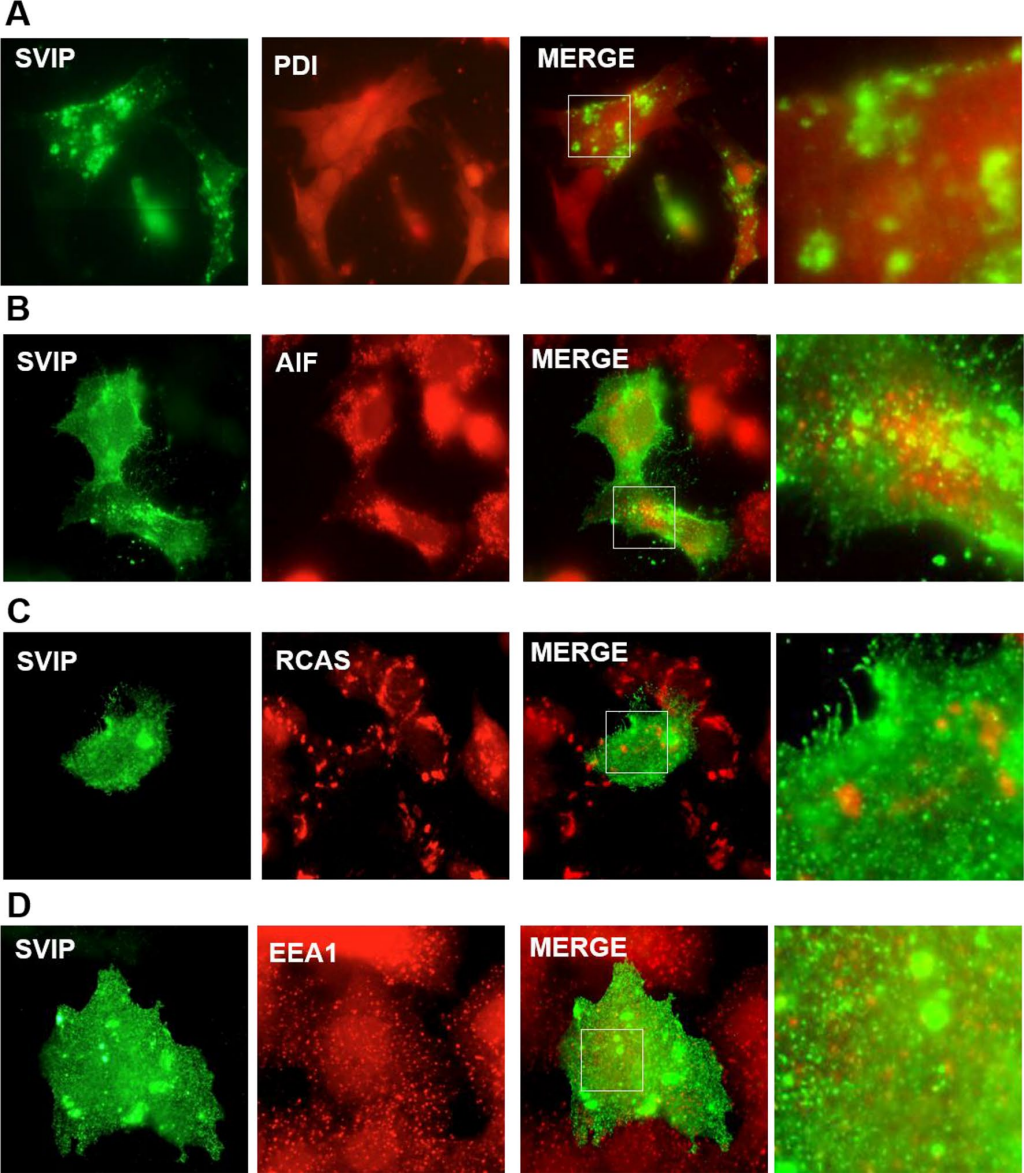

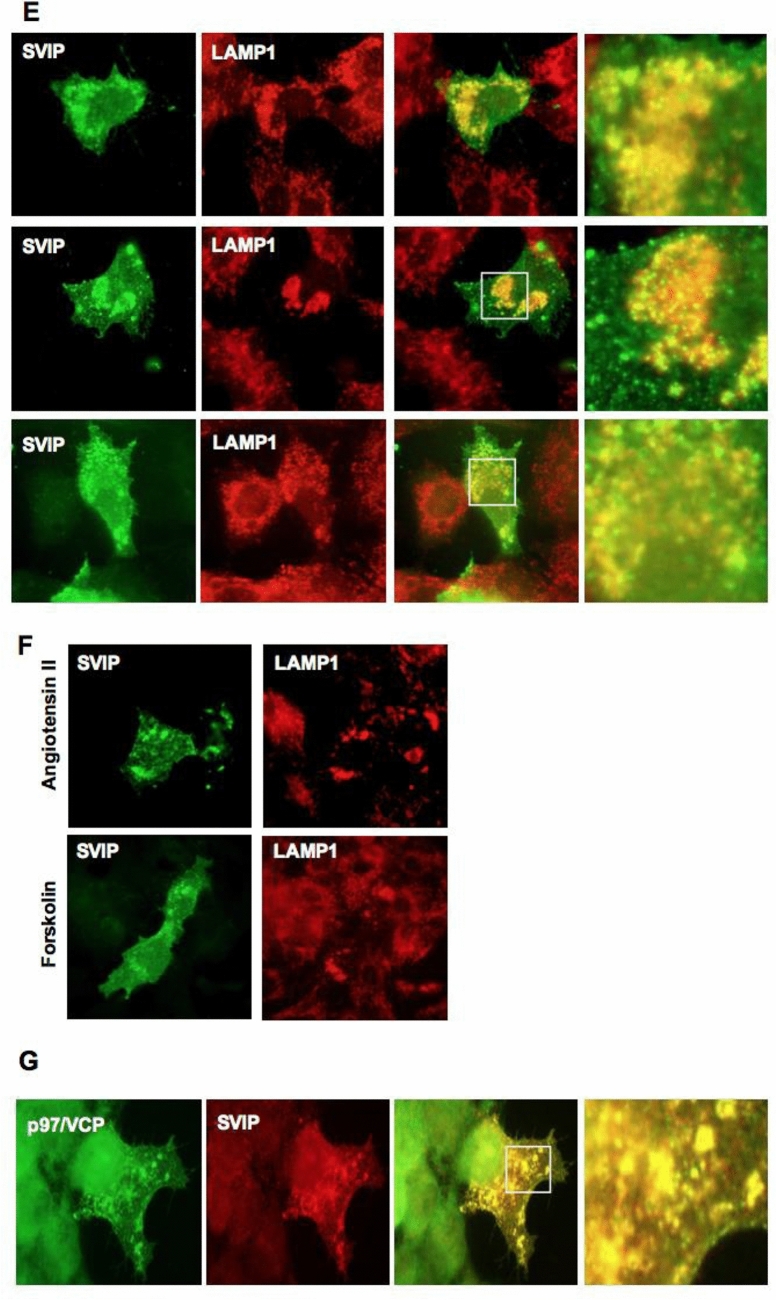
SVIP is co-localized with lysosomes and p97/VCP. H295R cells were transfected with 0.5 μg pCIneo-SVIP-His plasmid, then cells were co-stained with anti-His antibody and specific antibodies against organelle markers: (**A**) PDI (ER marker), (**B**) AIF (mitochondria marker), (**C**) RCAS (Golgi marker), (**D**) EEA1 (early endosomes marker), and (**E**) LAMP1 (lysosomal marker). (**F**) SVIP overexpressed cells were treated with 0.1 μM angiotensin II or 10 μM forskolin for 24 h and double immunostaining of SVIP and LAMP1 was performed. (**G**) SVIP relocalized p97/VCP. Images were taken after double immunostaining with anti-His antibody for SVIP and anti-VCP antibody for endogenous p97/VCP.

Next, we analyzed the co-localization of SVIP with well-defined organelle markers. Analysis of merged pictures obtained from double-labeling immunofluorescence showed no positive co-localizations of ectopic SVIP with the ER marker PDI (protein disulfide isomerase) (Fig. 4A), the mitochondria marker AIF (apoptosis-inducing factor) (Fig. 4B), the Golgi marker RCAS (receptor-binding cancer antigen expressed on SiSo cells) (Fig. 4C), and the early endosome marker EEA1 (early endosome antigen 1) (Fig. 4D). On the other hand, SVIP was found to be highly co-localized with the lysosome marker LAMP1 (lysosome-associated membrane protein-1) (Fig. 4E). Furthermore, LAMP1 is normally localized in vesicles throughout the cells, while in cells with high SVIP overexpression we observed lysosomal clustering in the juxtanuclear regions both in basal conditions (Fig. 4E) and when steroidogenesis was stimulated with forskolin and angiotensin II (Ang II) (Fig. 4F). Additionally, overexpression of SVIP caused cellular relocalization of p97/VCP in adrenal cortex cells (Fig. 4G).

### SVIP is downregulated by stimulators of steroidogenesis in H295R cells

**Figure 5:**
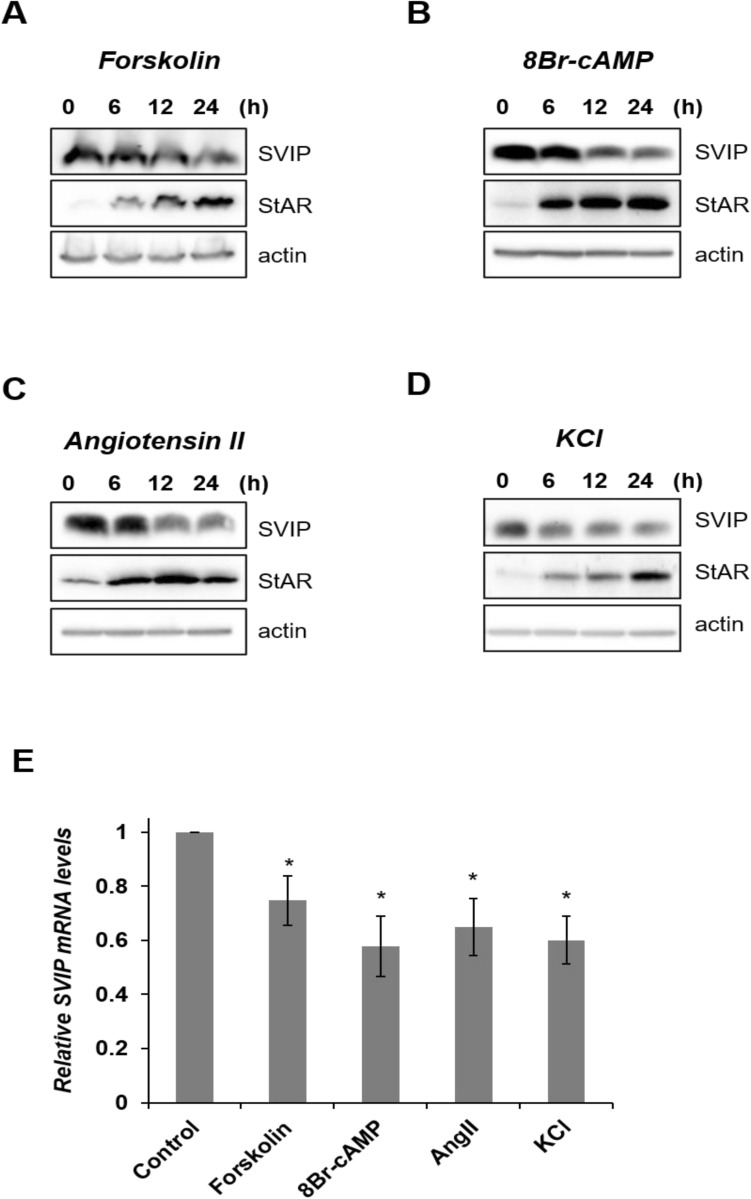

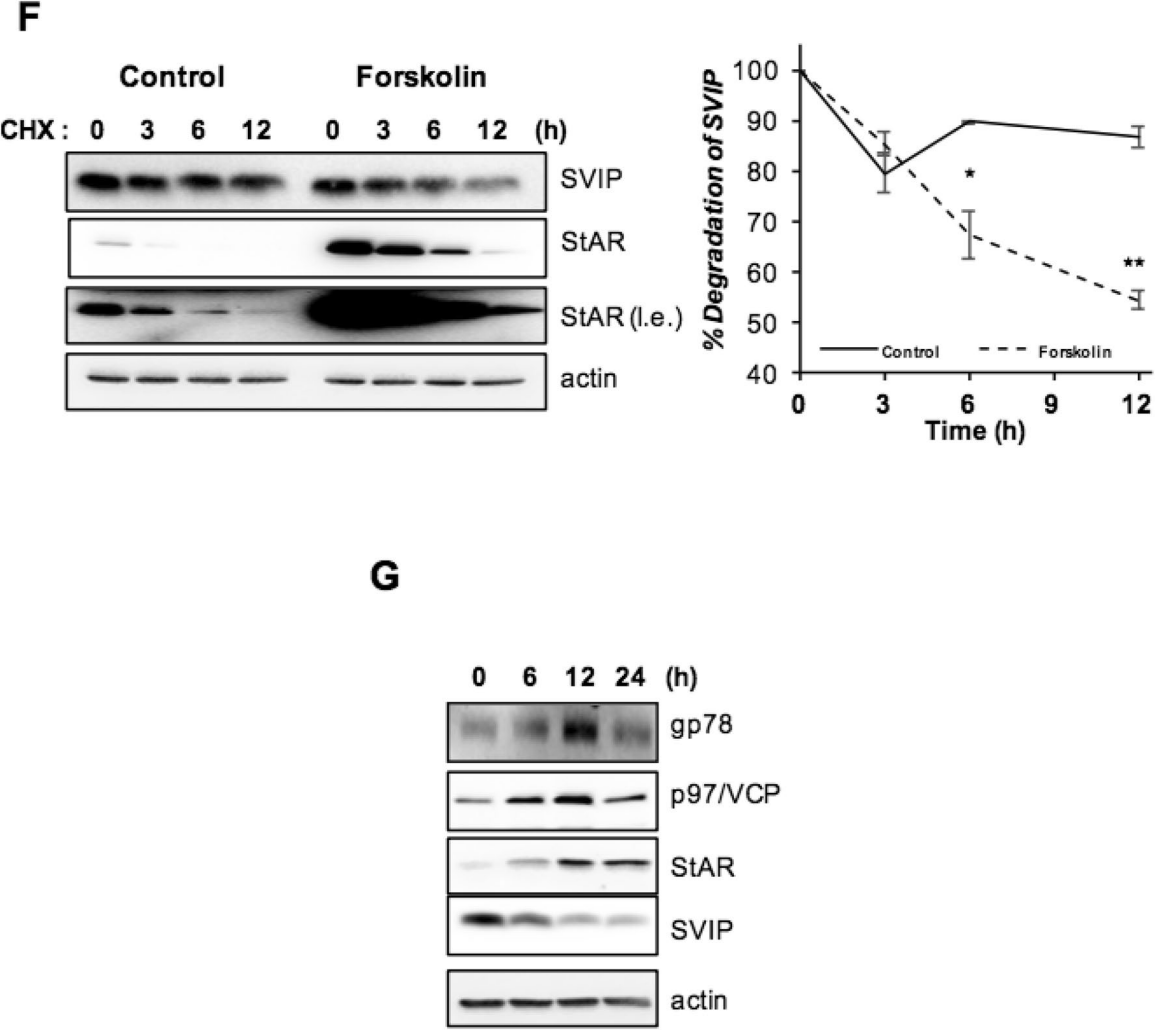
Stimulation of steroidogenesis downregulates SVIP. H295R cells were treated with (**A**) 10 μM forskolin, (**B**) 0.5 mM 8Br-cAMP, (**C**) 0.1 μM angiotensin II or (**D**) 14 mM KCl for the indicated times and SVIP protein levels were determined via immunoblotting (IB). Actin antibody was hybridized to the same membranes to verify equal protein loading. (**E**) SVIP mRNA was quantified by RT-qPCR. The experiment was repeated twice with at least three replicates. Error bars represent standard error (*p < 0.05). (**F**) The degradation of SVIP was analyzed by cycloheximide (CHX) chase on forskolin or vehicle-treated H295R cells. The SVIP and StAR levels were determined via IB (l.e.: longer exposure). Densitometric analysis of SVIP levels by ImageQuant is shown in the right panel (mean ± S.D., n = 3) (*p < 0.05 and **p < 0.01) (**G**) Following treatment with forskolin for the indicated times, expression levels of gp78, p97/VCP, SVIP, and StAR were detected by IB using antibodies against them. Actin was used as a loading control.

Forskolin or 8Br-cAMP treatment causes differentiation of H295R cells into “zona fasciculata-like cells”, which in turn increases the biosynthesis of cortisol and androgens via cAMP and the protein kinase A pathway^22^. Therefore, we incubated H295R human adrenocortical cells were incubated with 10 µM forskolin or 0.5 mM 8-Br-cAMP for increasing time periods up to 24 h and evaluated SVIP expression by immunoblotting. Both treatments decreased the levels of SVIP protein while upregulating StAR (steroidogenic acute regulatory protein) (Fig. 5A,B), a protein that shuttles cholesterol from the outer to the inner mitochondrial membranes in a rate-limiting step^23^. H295R cells can also be differentiated into more “glomerulosa-like cells” and synthesize more aldosterone via pre-treatment with angiotensin II (Ang II) or potassium chloride (KCl)^24^. Our results showed that both KCl and Ang II also decreased SVIP protein levels similar to forskolin or 8Br-cAMP (Fig. 5C,D).

In order to substantiate our findings and determine the mechanism underlying the downregulation of SVIP protein levels, we also evaluated the transcriptional regulation of SVIP by steroidogenic stimulators. Our data revealed that all tested stimulators diminished the SVIP mRNA compared to vehicle-treated control cells (Fig. 5E). Next, we carried out cycloheximide chase assay to determine whether SVIP protein stability is regulated by forskolin. Indeed, in addition to the downregulation of SVIP mRNA levels, forskolin treatment enhanced SVIP protein degradation (Fig. 5F). These findings indicate that steroidogenic stimulators reduce SVIP transcription as well as accelerate SVIP protein degradation.

Next, we sought to determine whether stimulating steroidogenesis could also alter the expression levels of ubiquitin ligase gp78 and retrotranslocation protein p97/VCP, two proteins reported to physically and functionally interact with SVIP. We found that forskolin treatment up to 12 h augmented expression of gp78 and p97/VCP, suggesting that steroidogenic stimulator treatment physiologically regulates the levels of the ERAD components (Fig. 5G).

### SVIP regulates cortisol and DHEA secretion via modulation of steroidogenesis-related protein levels

**Figure 6:**
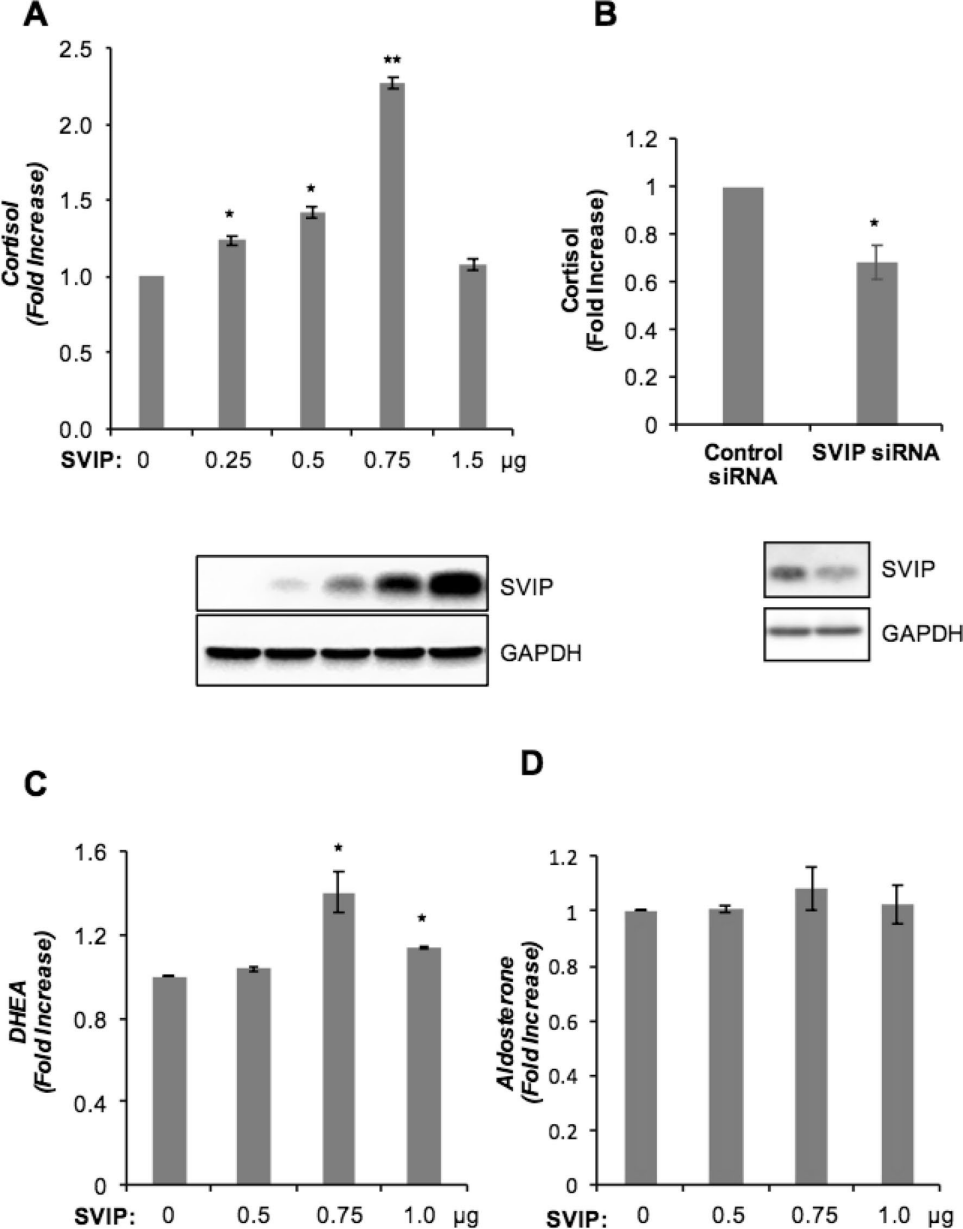
SVIP biphasically regulates cortisol and DHEA secretion in H295R cell line. (**A**) H295R cells were transfected with 0.25, 0.5, 0.75, and 1.5 μg SVIP. Six hours later, the cell culture media was replenished and both cells and cell culture supernatant were harvested after 48 h. The amount of cortisol in the growth media was quantified by ELISA. Cortisol amounts were normalized to the cellular protein concentration and presented as fold change compared to the cells that did not overexpress SVIP. The data graphed represent the mean ± SD. SVIP expression levels were determined by IB. GAPDH was used as loading control. (**B**) H295R cells were transfected with control or SVIP siRNA. At 24 h after transfection, fresh growth media was added. Both cells and cell culture supernatant were harvested 48 h later. Cortisol measurements and evaluation of SVIP expression levels were done as described for (**A**). (**C**,**D**) H295R cells were transfected with 0.5, 0.75, and 1.0 μg SVIP and processed as described for (**A**). The amount of DHEA (**C**) or aldosterone (**D**) in growth media was quantified by ELISA. Hormone levels were normalized to the cellular protein concentration and presented as fold change compared to cells that did not overexpress SVIP. The data graphed represent the mean ± SD (*p < 0.05, **p < 0.001, n = 3).

As SVIP levels were regulated by steroidogenic stimulators, we next investigated the effect of SVIP on steroid hormone secretion. Surprisingly, the cortisol secretion was significantly augmented in a dose-dependent manner up to certain SVIP overexpression level, but the highest tested SVIP level lost its ability to increase the cortisol levels (Fig. 6A). Concomitantly, when SVIP expression was decreased by *RNAi*, basal cortisol secretion was significantly diminished (Fig. 6B). We also investigated the effect of SVIP on other major steroid hormones such as aldosterone and DHEA. While aldosterone levels were not changed by SVIP overexpression (Fig. 6D), our data revealed that SVIP overexpression moderately enhanced DHEA biosynthesis (Fig. 6C). Furthermore, when the cumulative effect was investigated, SVIP was found to further enhance forskolin-stimulated cortisol biosynthesis (Supplementary Fig. 2). Together, these data suggest that SVIP regulates adrenal cortisol and DHEA pathways but not the aldosterone biosynthesis pathway.

**Figure 7:**
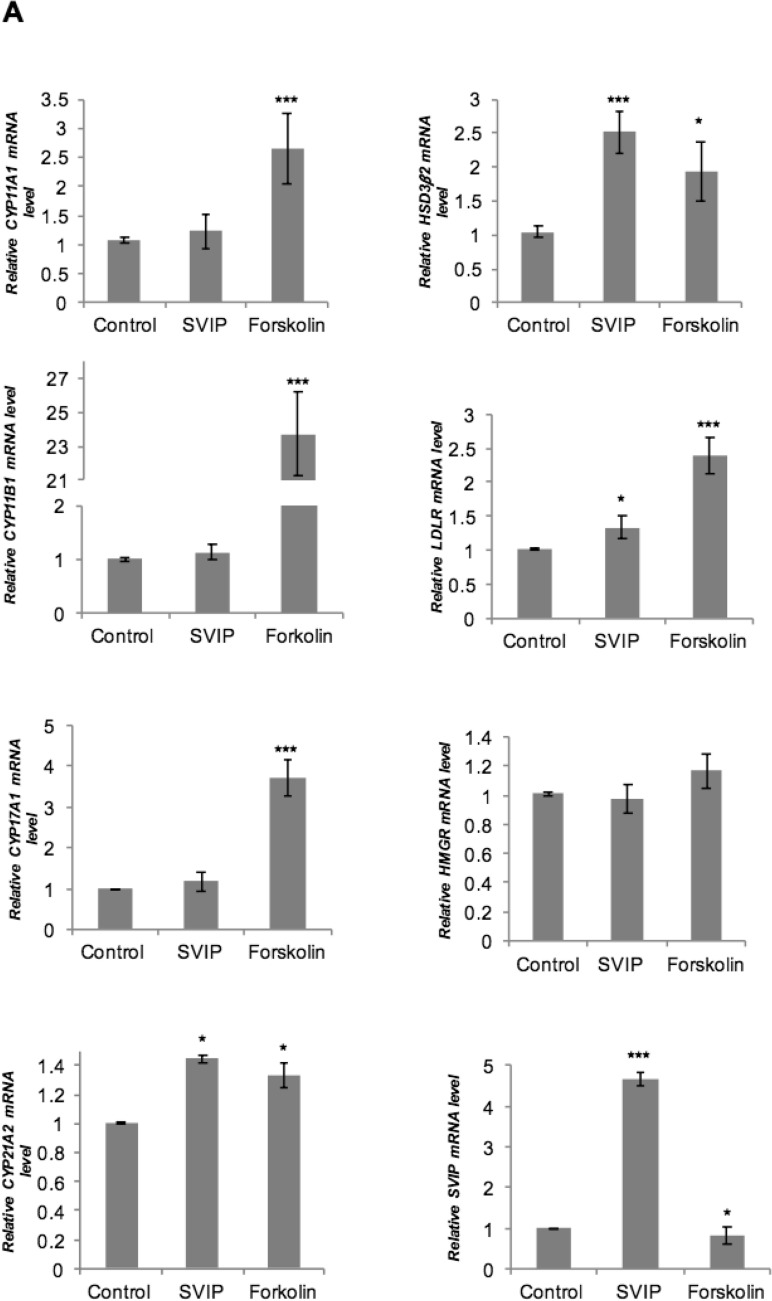

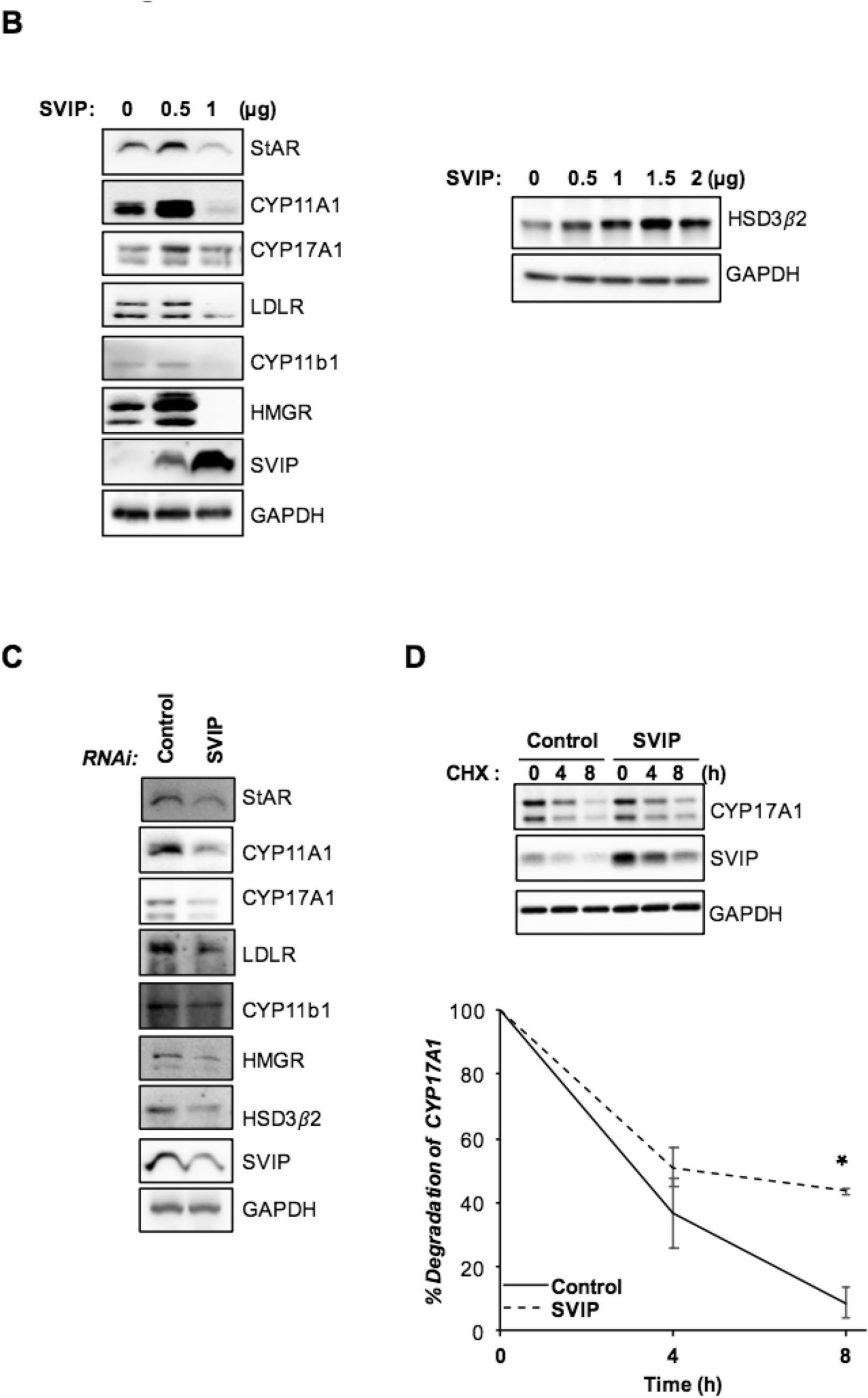
SVIP modulates the expression levels of several proteins related to steroidogenesis. (**A**) After H295R cells were transfected with 0.75 μg SVIP, mRNA levels of CYP17A1, CYP11A1, CYP11B1, HSD3β2, LDLR, HMGR, CYP21A2, and SVIP were examined by RT-qPCR. The experiment was repeated at least three times with three replicates. Error bars represent standard error (*p < 0.05 and ***p < 0.001) (**B** and **C**) Protein expression levels of CYP17A1, CYP11A1, CYP11B1, HSD3β2, LDLR, StAR, HMGR, and SVIP were determined by IB of cells transfected with (**B**) 0.5 and 1.5 μg plasmid encoding SVIP or (**C**) SVIP siRNA. (**D**) Cells were transfected with 1.5 μg SVIP and then treated with cycloheximide for the indicated times. CYP17A1 and SVIP levels were determined via IB. The degradation rates of substrates were calculated using three independent experiments (*p < 0.05). GAPDH was used as loading control.

Steroid hormones are essential signaling molecules that regulate multiple physiological processes. In adrenal steroidogenesis, the biosynthesis of cortisol and DHEA occurs from cholesterol via the concerted action of P450 heme-containing monooxygenases (CYPs) and 3β-hydroxysteroid dehydrogenase (3β-HSD) enzymes in adrenal cortex^25–27^. Furthermore, 3-hydroxy-3-methylglutary coenzyme A reductase (HMGR) and low-density lipoprotein receptor (LDLR) are equally important in assuring sufficient amounts of cholesterol for steroid hormone production^28,29^. Since the transcriptional regulation of these genes is highly studied, we first analyzed the effect of SVIP overexpression in mRNA expression levels of some key genes involved in cholesterol uptake, biosynthesis or mobilization and steroidogenesis. Overexpression of SVIP did not significantly change the mRNA levels of CYP11A1, CYP11B1, CYP17A1, or HMGR levels. LDLR and CYP21A2 mRNA levels were slightly increased, while HSD3β2 mRNA levels showed almost two-fold upregulation with SVIP overexpression (Fig. 7A).

SVIP has been reported to be involved in cellular protein degradation by regulating ERAD and autophagy^6,8,9^. Therefore, we also determined levels of the key proteins involved in steroid hormone biosynthesis. While a certain degree of SVIP overexpression consistently enhanced StAR, CYP11A1, CYP11B1, CYP17A1, HSD3β2, HMGR, and LDLR protein levels, its high overexpression resulted in a significant downregulation of all of these proteins except HSD3β2 (Fig. 7B), consistent with the increase in HSD3β2 mRNA levels observed previously (Fig. 7A). HSD3β2 protein level was found to be augmented even at higher SVIP overexpression level. Moreover, SVIP overexpression further enhanced forskolin-stimulated CYP17A1 and StAR levels even at higher concentrations, suggesting the high level of induction obtained with forskolin masks the inhibitory effect of high levels of SVIP (Supplementary Fig. 3). On the other hand, the protein levels of StAR, CYP11A1, CYP11B1, CYP17A1, HSD3β2, HMGR, and LDLR were significantly decreased by silencing SVIP in H295R cells (Fig. 7C), which is well correlated with cortisol secretion data (Fig. 6).

Since increased SVIP expression enhanced the levels of tested proteins but did not affect their transcription levels, except for HSD3β2, LDLR, and CYP21A2, we next investigated the effect of SVIP mainly on the degradation rate of CYP17A1, an enzyme that catalyzes the key branching point in the adrenal steroid hormone synthesis pathway towards cortisol and DHEA biosynthesis. Indeed, CYP17A1 degradation was found to be slowed even at high SVIP overexpression levels in the cycloheximide chase assay (Fig. 7D). Thus, our data showed that consistent with its role as ERAD inhibitor, SVIP overexpression decreased the turnover rate of CYP17A1. These results further suggest that the downregulation of proteins at high SVIP levels is not due to the enhanced protein degradation.

**Figure 8:**
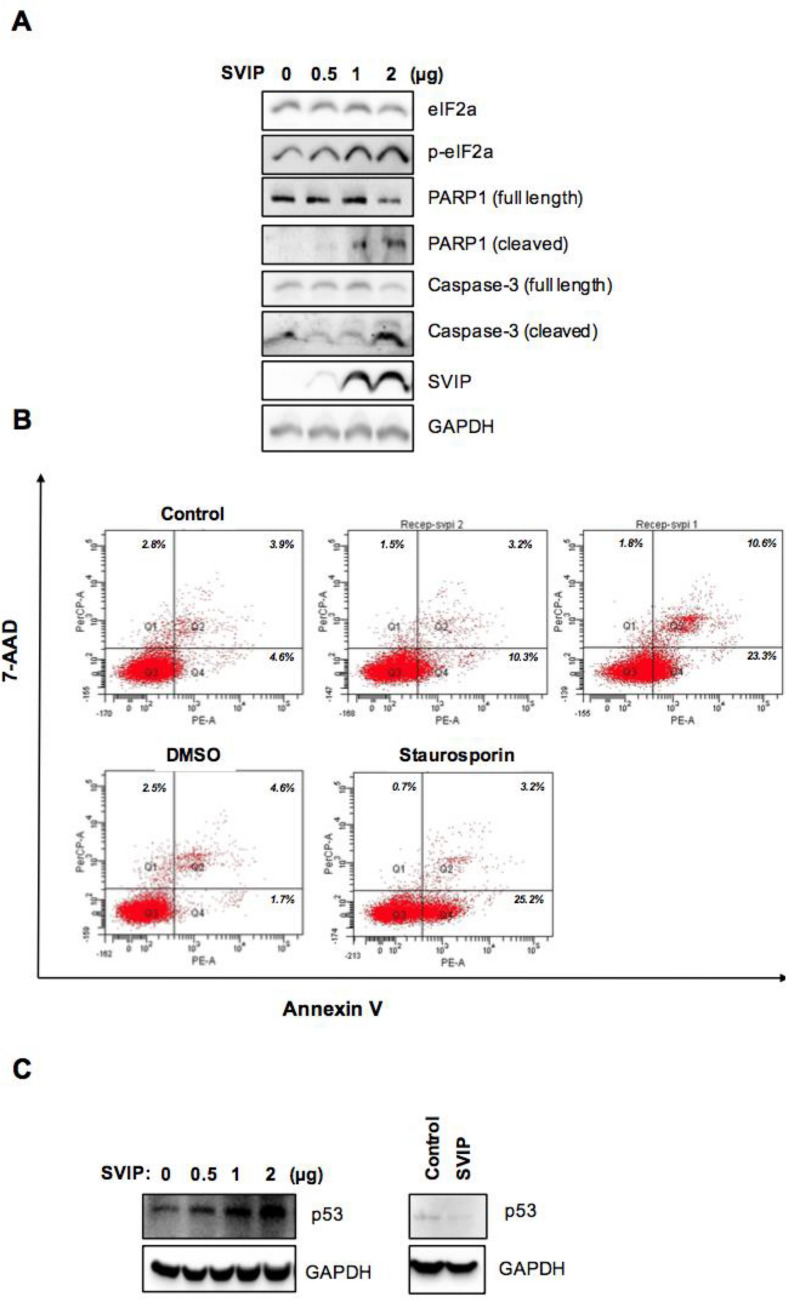
High expression of SVIP induces apoptosis in the H295R cell line. (**A**) H295R cells were transfected as indicated and harvested 24 h post-transfection. The expression levels of the proteins of interest were investigated using IB. (**B**) H295R cells were stained by Annexin V/7-AAD and analyzed via flow cytometry 24 h after transfection. Additionally, 1 μM staurosporine (Sta) was used to induce apoptosis and DMSO treatment was used as its control. (**C**) H295R cells transfected with plasmid encoding SVIP (left) or SVIP siRNA (right). The p53 protein levels were investigated in H295R cells by IB. GAPDH was used as loading control.

One possible explanation for the decreased protein levels in cells that express SVIP above a certain level might be global translational attenuation. Therefore, we next sought to investigate the phosphorylation of eIF2α, which is the best characterized mechanism for the regulation of translational initiation^30^. We observed that SVIP overexpression increased p-eIF2α levels in a dose-dependent manner (Fig. 8A). Furthermore, SVIP enhanced the levels of cleaved caspase-3 and PARP-1 levels implying that high SVIP expression promoted apoptosis in H295R cells (Fig. 8A). Apoptosis of cells transfected with SVIP expressing plasmid was significantly higher than that in vector-transfected control cells (Fig. 8B). Additionally, apoptotic morphology was observed in SVIP overexpressing cells (Supplementary Fig. 4). Because it was previously reported that SVIP as an ERAD inhibitor may delay the degradation of tumor suppressor p53^14^, which regulates the cell cycle and acts as guardian of genome stability, we next evaluated the effect of SVIP on the p53 levels in H295R cells and found that p53 levels were increased with SVIP overexpression and decreased with SVIP silencing (Fig. 8C). In summary, our data suggest that exaggerated SVIP expression levels triggers caspase-dependent apoptosis along with an increase in eIF2α phosphorylation of and p53 abolishment in H295R cells.

## Discussion

ERAD is a well-characterized protein quality control mechanism functioning in the degradation of misfolded ER proteins. It also plays a role in the destruction of some properly folded proteins, the best-known example being HMGR, which is the key enzyme of the sterol biosynthesis pathway^31–33^. The degradation of HMGR by ERAD is a vital feedback inhibition system for sterol homeostasis both in yeast and mammalian cells. Several ERAD ubiquitin ligases such as gp78, TRC8, Hrd1, March6, and RNF145 have been demonstrated to play a role in the turnover of HMGR^34,35^. Besides HMGR, other proteins involved in sterol biosynthesis, such as Insig proteins and squalene monooxygenase are reported to be ERAD substrates^32,36^. It has also indicated that gp78 mediates the degradation of apolipoprotein B100, one of the main LDL-VLDL lipoproteins involved in cholesterol transport^37^. Further evidence that ERAD has a conserved role in sterol regulation is the biosynthesis of sterols and sterol-derived metabolites in plants^38^. Therefore, in addition to protein quality control, ERAD has emerged as a key mechanism that controls the flux of metabolic pathways by altering the abundance of sterol biosynthetic enzymes and their regulators. Considering its role in lipid and protein homeostasis, tight regulation of ERAD is critical for cells. Although there are several diverse ERAD branches, they all converge on the cytosolic p97/VCP ATPase complex, which extracts ubiquitinated substrates from the ER membrane and delivers them to the proteasome for degradation^39^. Therefore, SVIP is notable as an ERAD component for being the first identified endogenous inhibitor of ERAD through its interaction with p97/VCP.

As the adaptor protein for multifunctional p97/VCP, SVIP has also been shown to be involved in multiple cellular processes such as the regulation of vacuole formation, autophagy, and ERAD inhibition^4,6,8^. In an SVIP expression screening assay, we showed that unlike its gradually increasing expression in the developing nervous tissues such as cerebrum, cerebellum, sciatic nerve, and medulla spinalis, SVIP expression in the adrenal gland remained at a consistently high level during postnatal development, suggesting that SVIP may function throughout the developmental process of the adrenal gland. Our immunohistochemistry and double immunofluorescence data further demonstrated that SVIP expression is not only organ specific but also differs between the regions of the rat adrenal gland, where SVIP is exclusively expressed in the adrenal cortex (Fig. 2). Since the adrenal cortex was the only tissue found to express SVIP at all developmental stages, we aimed to analyze the role of SVIP in adrenal function. Importantly, there are significant differences between rodent and human adrenal physiology, mainly in terms of the zonation and the repertoire of steroidogenic enzymes^19,20^. Thus, to further study the role and regulation of SVIP in adrenal cortex cells, we utilized the H295R pluripotent adrenocarcinoma cell line, one of the best characterized cellular models for studying adrenal cortex cell biology. This cell line expresses genes that encode all the key enzymes for steroidogenesis, having the physiological characteristics of zonally undifferentiated human adrenal cells and producing all of the steroid hormones found in the adult adrenal cortex^24^. We observed that SVIP localized as punctuated structures both in rat adrenal cortex cells and H295R cells (Fig. 3). Furthermore, we demonstrated that the expression of ectopic SVIP displays co-localization with lysosomal membrane protein LAMP1 and causes lysosomal clustering (Fig. 4). As shown previously in HeLa cells^8^, overexpression of SVIP relocalizes p97/VCP from the cytosol to the juxtanuclear vacuoles in H295R cells as well. Very recently, *Drosophila* SVIP was also shown to recruit p97/VCP to lysosomes in muscle cells^40^.

Adrenal steroidogenesis is a tightly regulated dynamic process, as adrenal steroid hormones are key regulators of a wide variety of physiological functions including blood pressure, inflammation, glucose metabolism, and stress^41–43^. Pre-synthesized hormones are not stored for immediate release and de novo biosynthesis of adrenal steroid is controlled by multiple regulatory mechanisms (e.g., transcriptional, post-translational and substrate transportation)^44^. Downregulation of SVIP both at the transcriptional and post-translational levels by steroidogenic stimulating agents in H295R cells suggests that SVIP may have a role in the steroid hormone biosynthesis pathway. However, when the effect of SVIP on steroid hormone levels was determined, SVIP overexpression up to certain levels caused an increase in cortisol and DHEA secretion while SVIP silencing diminished cortisol levels. This unprecedented result may suggest that SVIP plays a critical role in the termination of intracellular signaling events triggered by steroidogenic stimulators.

Besides steroidogenic enzymes, proteins involved in cholesterol biosynthesis and uptake are also regulatory factors of steroid hormone production^45,46^. Our data clearly demonstrate that the change in cortisol secretion capability of H295R cells via SVIP expression levels is highly correlated with the modulation of the protein levels such as StAR, HMGR, and CYP17A1. The downregulation of SVIP by steroidogenic stimulators and diminished levels of key proteins accompanied by a decrease in cortisol secretion by silencing of SVIP expression provide further evidence of the involvement of SVIP in the termination of steroid hormone biosynthesis signals in adrenal cells to prevent exaggerated steroidogenesis. As a glucocorticoid, cortisol biosynthesis is regulated by adrenocorticotropin (ACTH) in addition to the general regulatory mechanism. As cortisol is an overall catabolic hormone, excess cortisol reduces lean body mass, and muscle mass, induces insulin resistance, and may increase energy expenditure. Moreover, Cushing's syndrome is one of the well-known physiological disorders associated with excessive cortisol biosynthesis^47^. Thus, negative regulation of cortisol biosynthesis is important, and several negative feedback mechanisms have been reported, such as hypothalamic–pituitary–adrenal axis activity and decreased ACTH secretion via cortisol^48,49^. Nevertheless, our findings strongly suggest that modulation of SVIP expression may function as a novel regulatory mechanism of adrenal steroid biosynthesis.

Notably, we also found that the regulation of SVIP protein levels is extremely critical in adrenal cortex, where at immense overexpression of SVIP led to reduced levels of steroidogenic proteins along with diminished cortisol production (Fig. 7A). Our findings that SVIP at this high concentration still decreased the degradation rate of CYP17A1, which catalyzes the key branching point in adrenal steroid hormone synthesis pathway towards cortisol and DHEA biosynthesis (Fig. 7D), proposed that the opposite steroidogenic response at high SVIP levels was not directly linked to regulation of the degradation rate of tested proteins. Therefore, we hypothesized that overexpression of SVIP at high levels might impose stress on the ER via ERAD inhibition, causing the accumulation of unfolded or misfolded proteins in the ER lumen. To restore the normal ER functions, cells launch the Unfolded Protein Response (UPR), which primarily involves attenuating general protein synthesis, increasing the lumenal folding capacity, and increasing the degradation of misfolded proteins through ERAD or autophagy to resolve the ER overload^50,51^. In response to ER stress, cells attenuate global translation through PERK-mediated eIF2α phosphorylation^52^. Our results also demonstrated that exaggerated expression of SVIP significantly increased levels of p-eIF2α, which functions in translational attenuation during ER stress. Therefore, we speculate that the downregulation of the tested steroidogenic proteins at high SVIP levels may be due to inhibition of translation, but further experiments such as metabolic pulse labeling of newly synthesized proteins are required to confirm the relation between SVIP and translational attenuation in adrenal cortex cells.

When ER stress is not resolved, the prolonged UPR induces apoptosis to remove the stressed cells from the organism^53^. Interestingly, prolonged ER stress associated with the accumulation of misfolded proteins in the ER was shown to significantly upregulate SVIP^6^. It has also been shown that overexpression of SVIP increased the levels of tumor suppressor p53, which regulates the cell cycle and leads to apoptosis^14^. Our data also indicated that high SVIP levels promotes apoptosis in H295R cells. Considering a previous report indicating that mitochondrial dysfunction contributes to the decline of steroidogenesis in human granulosa cells^54^, it is plausible to speculate that SVIP-mediated induction of apoptosis may also decrease cortisol synthesis via apoptosis-related mitochondrial dysfunction.

Considering the role of ubiquitin ligase gp78 in ERAD and cholesterol biosynthesis together with its functional inhibition by SVIP, it is highly possible that gp78 may have a role in steroid hormone biosynthesis. Therefore, it is essential to further investigate the regulation of adrenal steroidogenesis by ERAD and its interplay with the activities of other organelles (such as mitochondria), since the biosynthesis of cortisol requires the activities of enzymes between the ER and mitochondria. Interestingly, p97/VCP functions in the extraction of ubiquitinated proteins from the outer mitochondrial membrane and presents ubiquitinated proteins to the proteasome for degradation^55^. Therefore, by removing p97/VCP from its functional complex, SVIP may also affect mitochondrial protein degradation. Proteomics and metabolomics analyses utilizing SILAC assay have consistently pointed out that epigenetic loss of SVIP is associated with depletion of some mitochondrial enzymes and oxidative respiration activity which reverts upon SVIP restoration^15^.

In conclusion, our results suggest that modulation of SVIP expression alters not only the expression levels of steroidogenic genes and hormone output, but also the expression of genes required for de novo cholesterol biosynthesis, uptake and trafficking. Furthermore, SVIP constitutes a double-edged sword in adrenal steroidogenesis: on one hand, SVIP positively regulates steroid hormone biosynthesis, while on the other, excessive SVIP expression induces apoptosis. Our study may suggest a key link between ERAD and sterol biology by providing evidence of the role of SVIP in adrenal hormone biosynthesis.

## Supplementary Information

Supplementary Figures.

## Data Availability

The data that support the findings of this study are available from the corresponding author (PBK) upon reasonable request.

## Abbreviations

3β-HSD: 3β-Hydroxysteroid dehydrogenase
AIF: Apoptosis-inducing factor
Ang II: Angiotensin II
ACTH: Adrenocorticotropin
CYPs: P450 heme-containing monooxygenases
EEA1: Early endosome antigen 1
ERAD: Endoplasmic reticulum-associated degradation
ER: Endoplasmic reticulum
HMGR: 3-Hydroxy-3-methylglutary coenzyme A reductase
IB: Immunoblotting
IHC: Immunohistochemistry
KCl: Potassium chloride
LAMP1: Lysosome associated membrane protein-1
LDRL: Low-density lipoprotein receptor
MBP: Myelin basic protein
OMM: Outer mitochondrial membrane
PDI: Protein disulfide isomerase
RCAS: Receptor binding cancer antigen expressed on SiSo cells
S.D: Standard deviation
StAR: Steroidogenic acute regulatory protein
SVIP: Small VCP-interacting protein
UPR: Unfolded protein response
ZF/R: Zona fasciculata/reticularis
