# Supplementary material for: Novel regulation mechanism of adrenal cortisol and DHEA biosynthesis via the endogen ERAD inhibitor small VCP-interacting protein
Source: Sci Rep. 2022 Jan 18;12:869. doi: 10.1038/s41598-022-04821-y (PMC8766438; doi:10.1038/s41598-022-04821-y)
Supplement: Supplementary file 1 — Supplementary Figures. [file 41598_2022_4821_MOESM1_ESM.pdf]

Supplementary Data

Novel regulation mechanism of adrenal cortisol and DHEA biosynthesis via the endogen ERAD inhibitor small VCP- interacting protein

Recep Ilhan[1], Göklem Üner[2], Sinem Yilmaz[3,4], Esra Atalay Sahar[3], Sevil Cayli[5], Yalcin Erzurumlu[1,#], Oguz Gozen[6], Petek Ballar Kirmizibayrak [1,3*]

[1] Department of Biochemistry, Faculty of Pharmacy, Ege University, 35100 Bornova-Izmir, Turkey

[2] Department of Bioengineering, Izmir Institute of Technology, 35430 Urla-Izmir, Turkey

[3] Department of Biotechnology, Graduate School of Natural and Applied Sciences, Ege University, Izmir, Turkey

[4] Department of Bioengineering, Faculty of Engineering, University of Alanya Aladdin Keykubat, Antalya, Turkey

[5] Ankara Yıldırım Beyazıt University, Medical Faculty, Department of Histology and Embryology, Ankara, Turkey

[6] Department of Physiology, School of Medicine, Ege University, Izmir, Turkey.

[*]Corresponding author: petek.ballar@ege.edu.tr

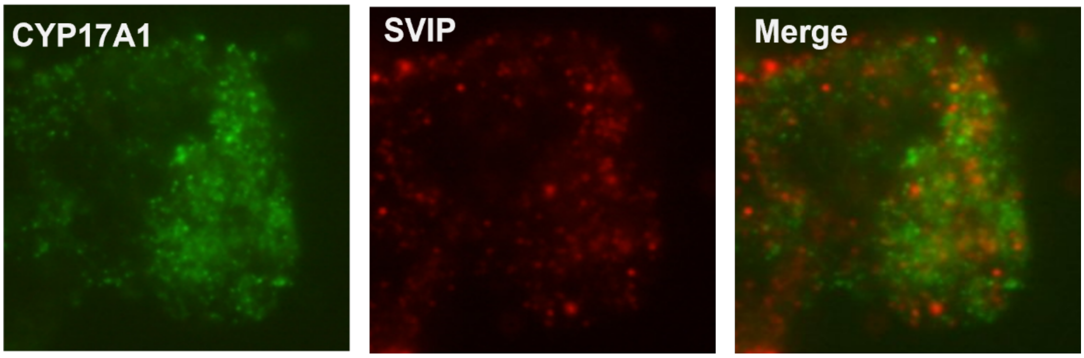

Supplementary Figure 1. SVIP and CYP17A1 do not co-localized. H295R cells were transfected with 0.25 µg pCIneo-SVIP-His plasmid. Then cells were co-stained with anti-His antibody and specific antibodies against CYP17A1.

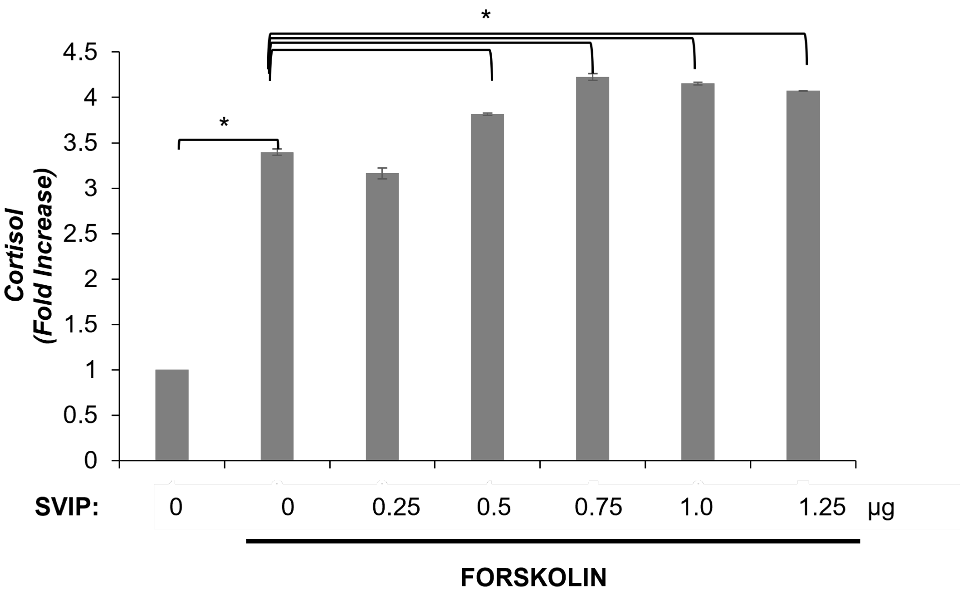

Supplementary Figure 2. Cumulative effect of SVIP and Forskolin on cortisol secretion in H295R cell line. H295R cells were transfected with 0.25, 0.5, 0.75, 1.0 and 1.5 µg SVIP. Then cells were treated with Forskolin. The amount of cortisol in growth media was quantified by ELISA. Cortisol amounts were normalized to the cellular protein concentration and presented as fold change compared to the cells that do not overexpress SVIP. Data graphed represent the mean ± SD.

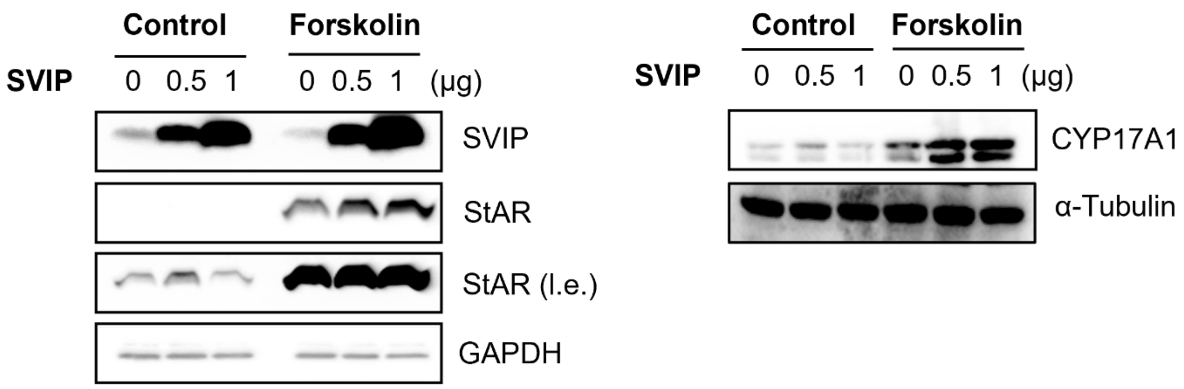

Supplementary Figure 3. Cumulative effect of SVIP and Forskolin on StAR and CYP17A1 in H295R cell line. After H295R cells were transfected with 0.5 and 1 μg SVIP, cells were treated with Forskolin or vehicle. The protein expression levels of CYP17A1, StAR, and SVIP were determined by IB using antibodies raised against them.

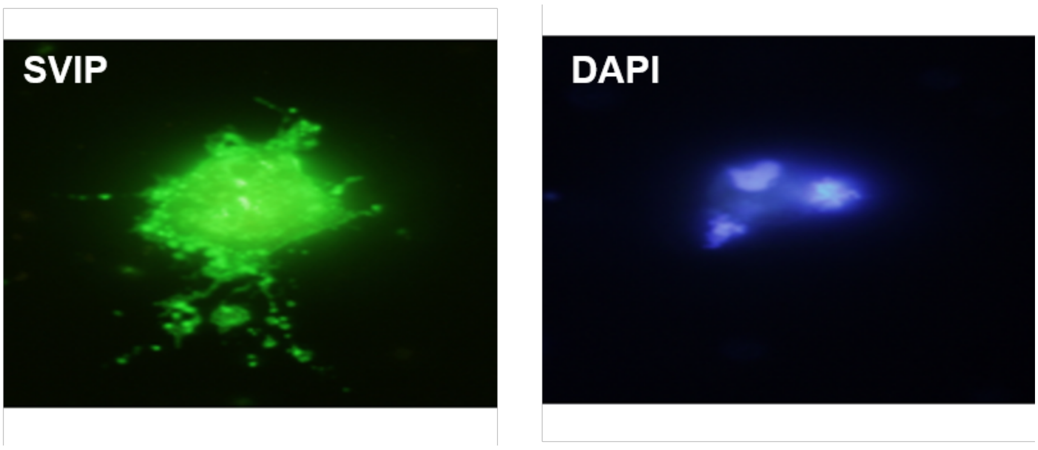

Supplementary Figure 4. SVIP mediated cell death have apoptotic morphology. Nucleus of H295R cells, which were transfected with 2 μg SVIP plasmid, were stained with DAPI.

A

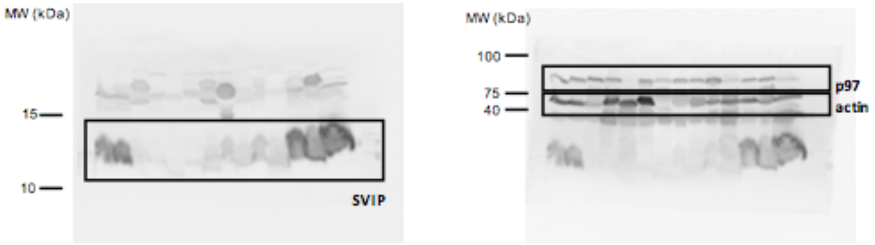
MW (kDa)
15
10
SVIP
MW (kDa)
100
75
40
p97
actin

B

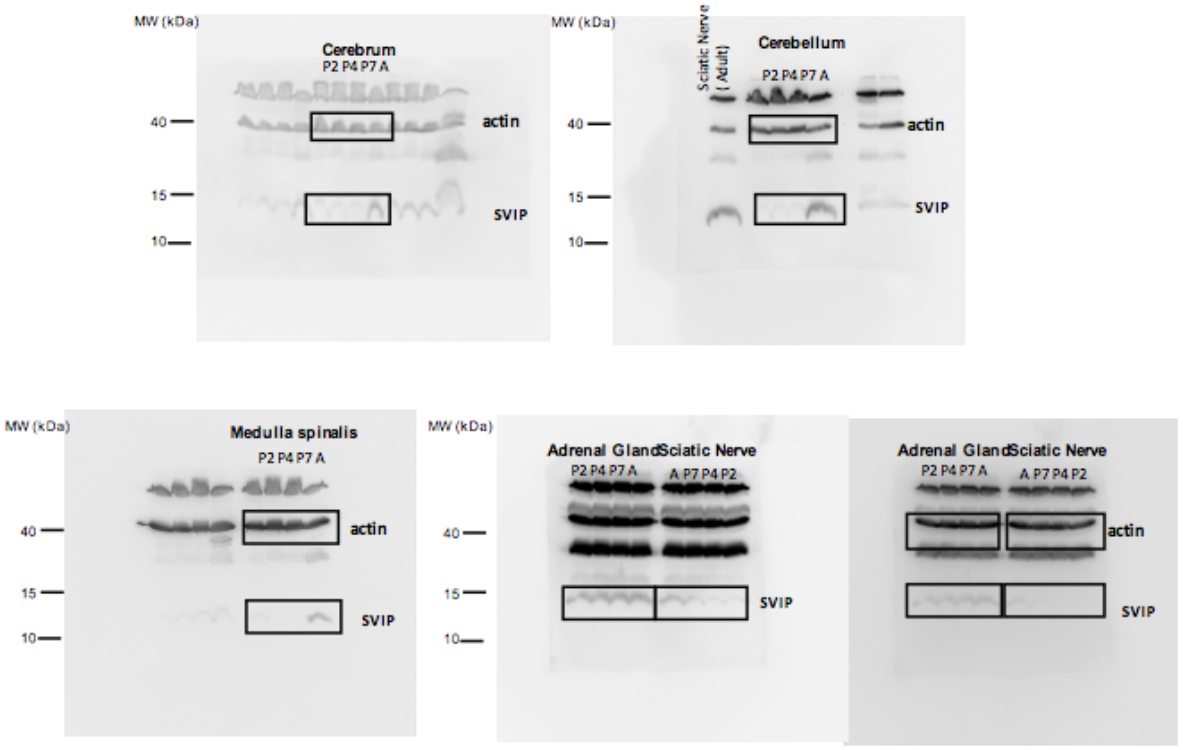
MW (kDa)
Cerebrum
P2 P4 P7 A
40
15
10
actin
SVIP
MW (kDa)
Sciatic Nerve (Adult)
Cerebellum
P2 P4 P7 A
40
15
10
actin
SVIP
MW (kDa)
Medulla spinalis
P2 P4 P7 A
40
15
10
actin
SVIP
MW (kDa)
Adrenal Gland
Sciatic Nerve
P2 P4 P7 A
A P7 P4 P2
40
15
10
SVIP
Adrenal Gland
Sciatic Nerve
P2 P4 P7 A
A P7 P4 P2
actin
SVIP

C

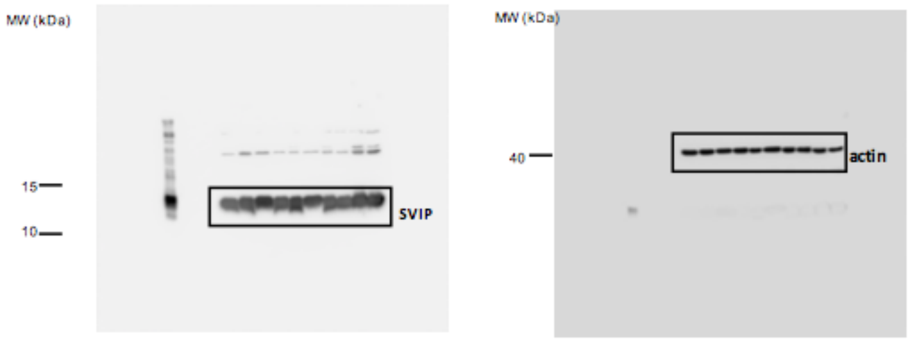
MW (kDa)
15
10
SVIP
MW (kDa)
40
actin

D

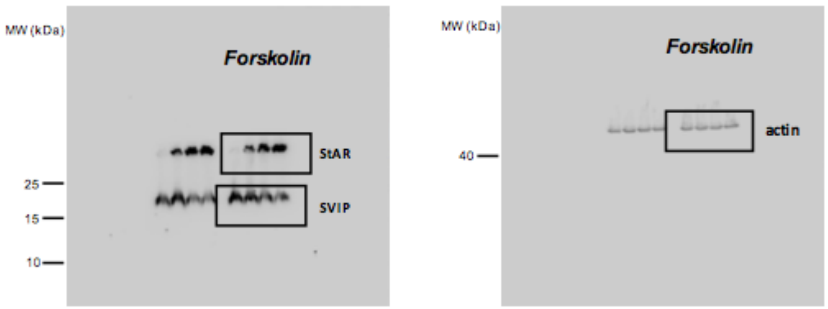
MW (kDa)
Forskolin
StAR
SVIP
25
15
10
MW (kDa)
Forskolin
actin
40

E

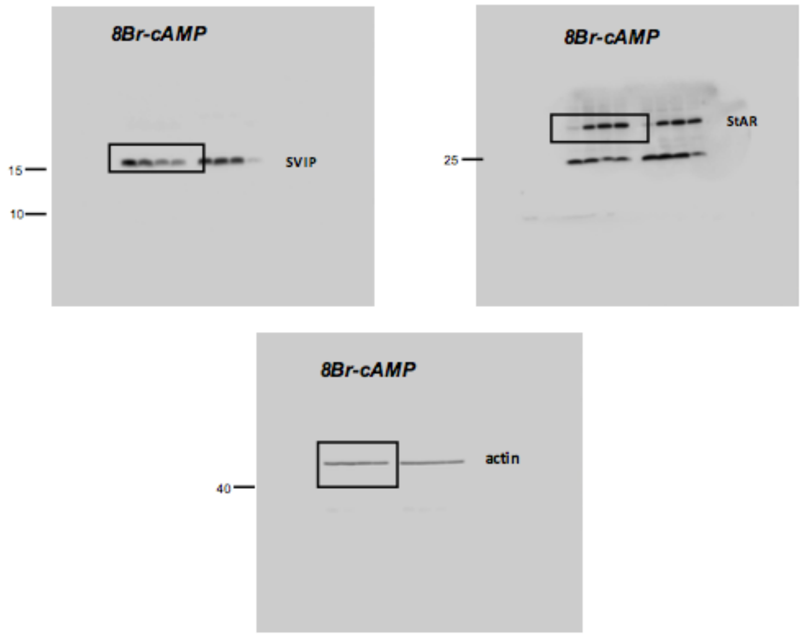
8Br-cAMP
SVIP
15
10
8Br-cAMP
StAR
25
8Br-cAMP
actin
40

F

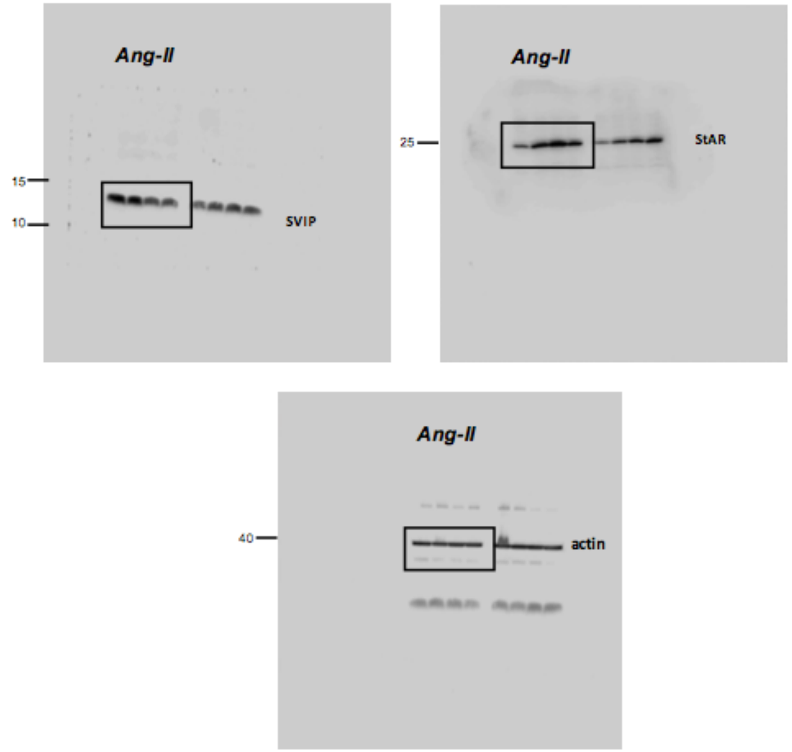
Ang-II
15
10
SVIP
Ang-II
25
StAR
Ang-II
40
actin

G

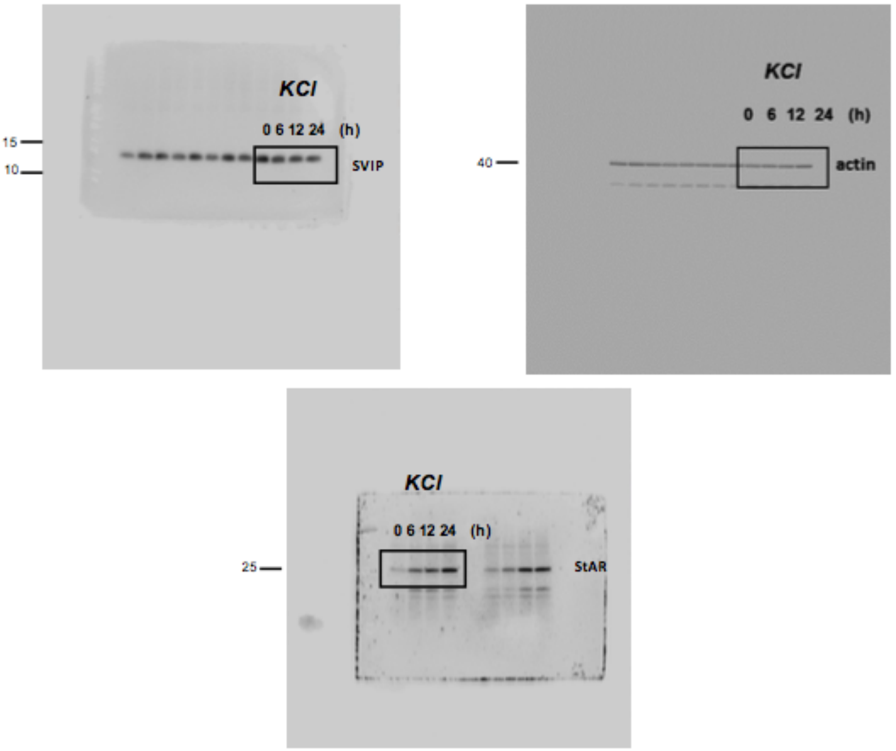
KCl
0 6 12 24 (h)
15
10
SVIP
KCl
0 6 12 24 (h)
40
actin
KCl
0 6 12 24 (h)
25
StAR

H

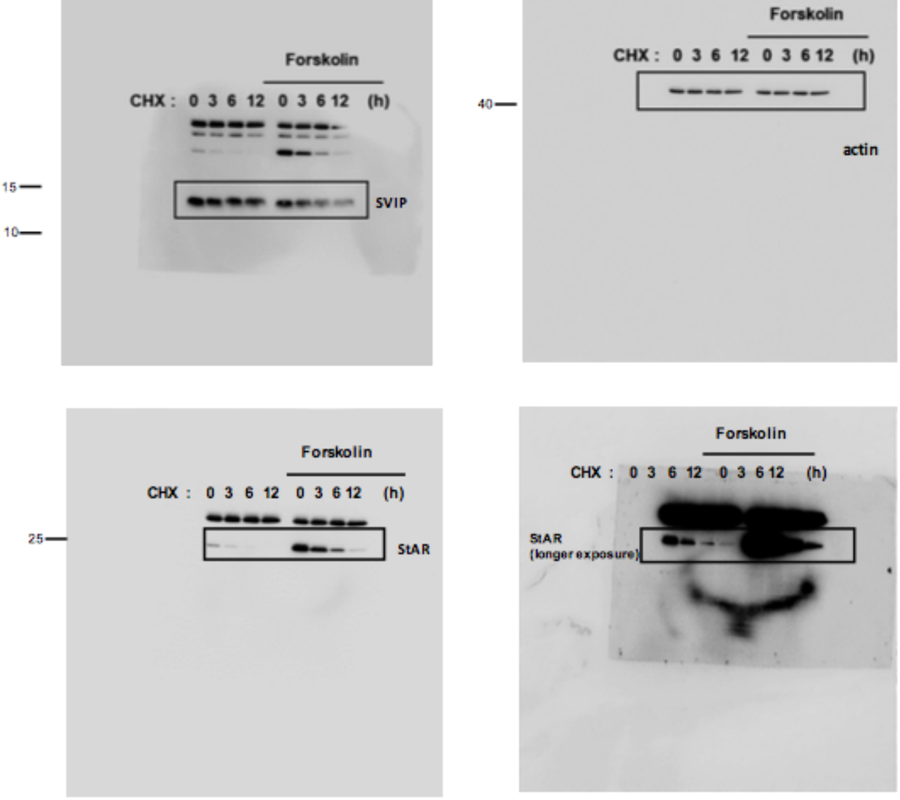
Forskolin
CHX : 0 3 6 12 0 3 6 12 (h)
15
10
SVIP
Forskolin
CHX : 0 3 6 12 0 3 6 12 (h)
40
actin
Forskolin
CHX : 0 3 6 12 0 3 6 12 (h)
25
StAR
Forskolin
CHX : 0 3 6 12 0 3 6 12 (h)
StAR
(longer exposure)

I

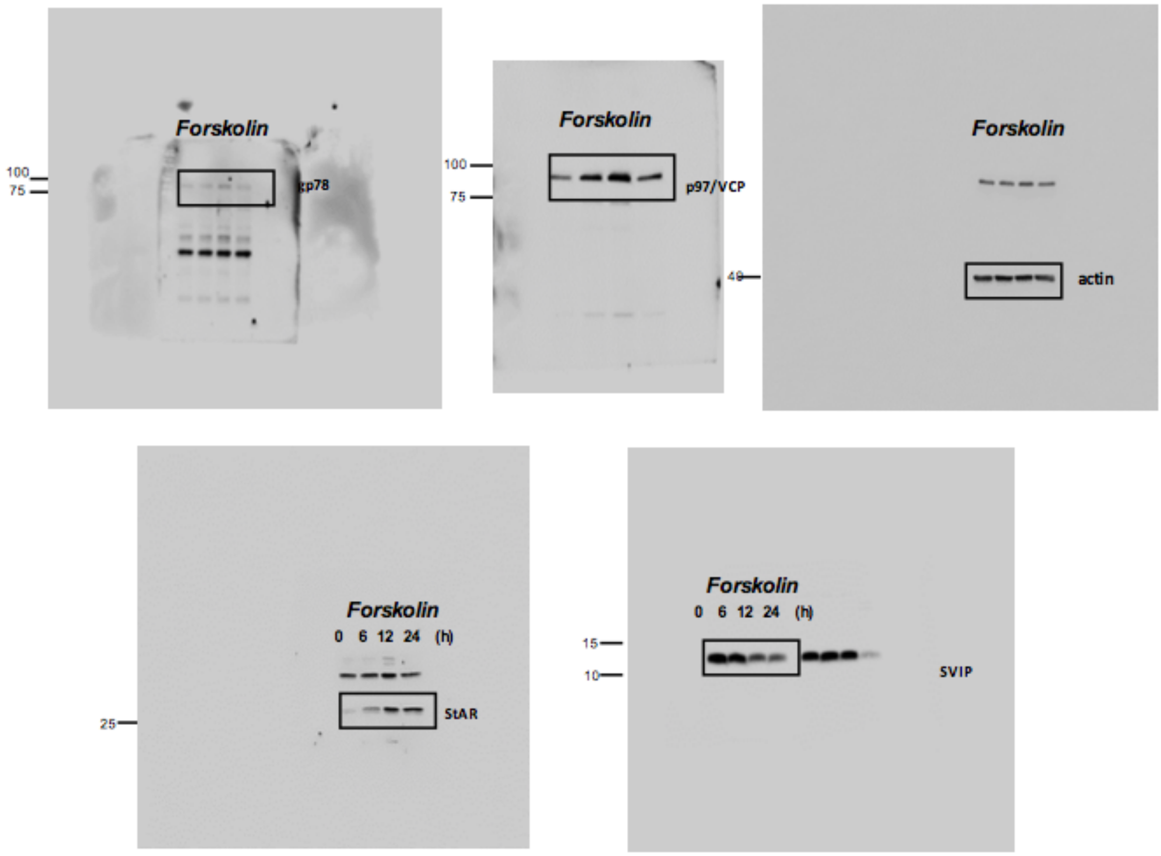

Forskolin
100
75
gp78
Forskolin
100
75
p97/VCP
Forskolin
40
actin
Forskolin
0 6 12 24 (h)
25
StAR
Forskolin
0 6 12 24 (h)
15
10
SVIP

J

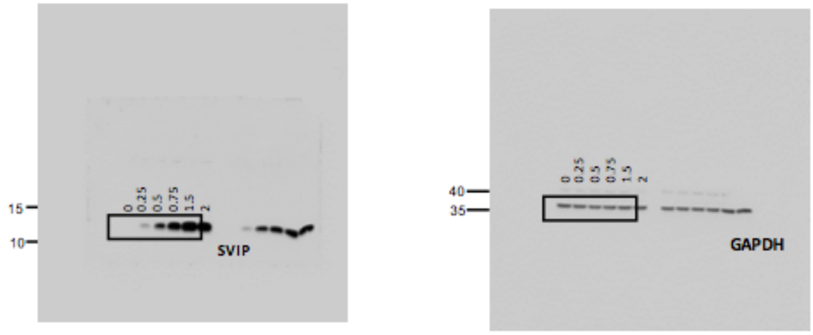

0
0.25
0.5
0.75
1.5
2
15
10
SVIP
0
0.25
0.5
0.75
1.5
2
40
35
GAPDH

K

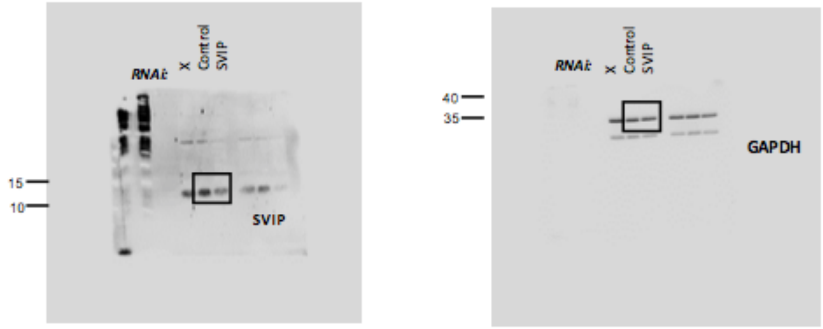

RNAi:
X
Control
SVIP
15
10
SVIP
RNAi:
X
Control
SVIP
40
35
GAPDH

L

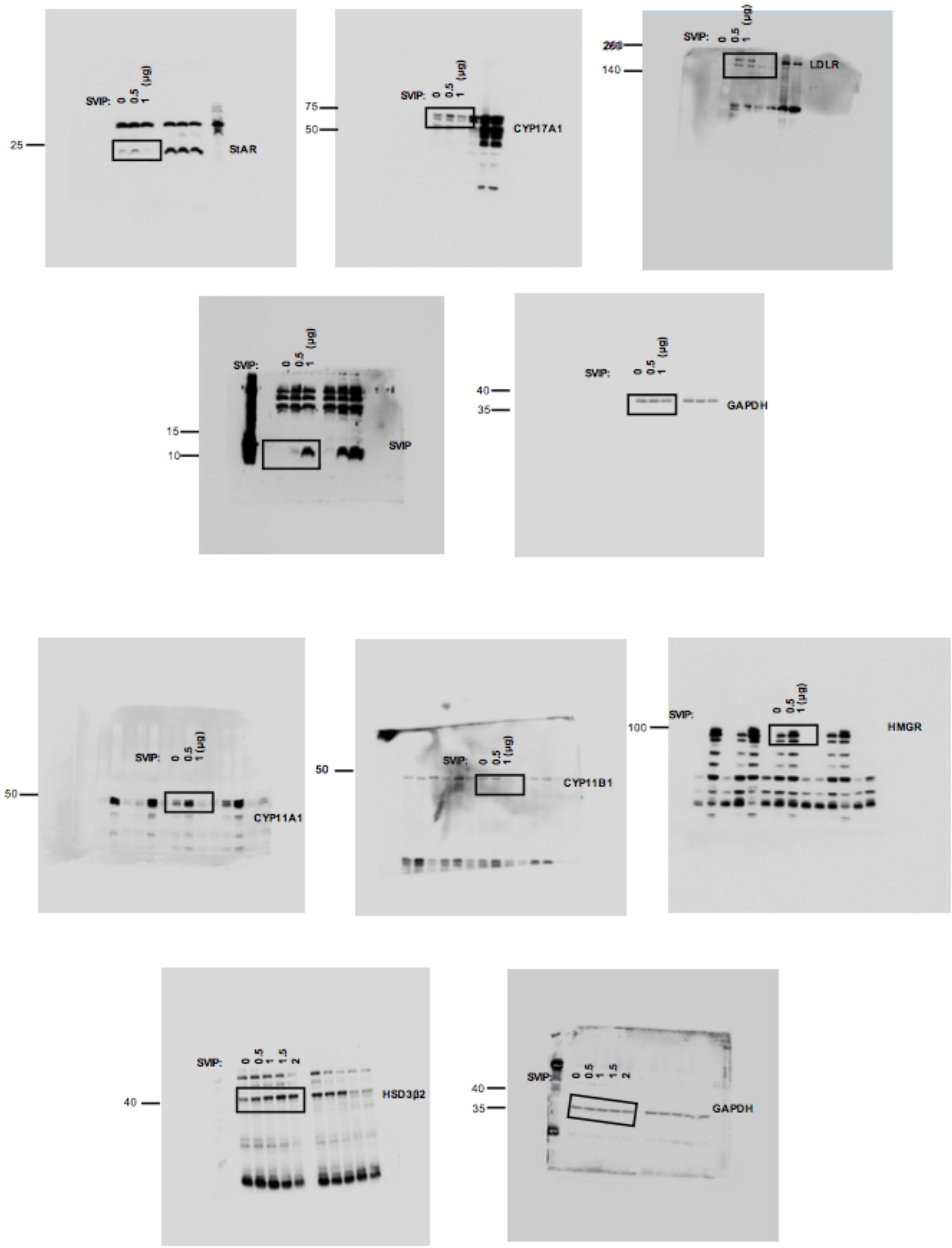
SVIP: 0 0.5 1 (μg)
25
StAR
75
50
CYP17A1
260
140
LDLR
15
10
SVIP
40
35
GAPDH
50
CYP11A1
CYP11B1
100
HMGR
SVIP: 0 0.5 1 1.5 2
40
HSD3β2
40
35
GAPDH

M

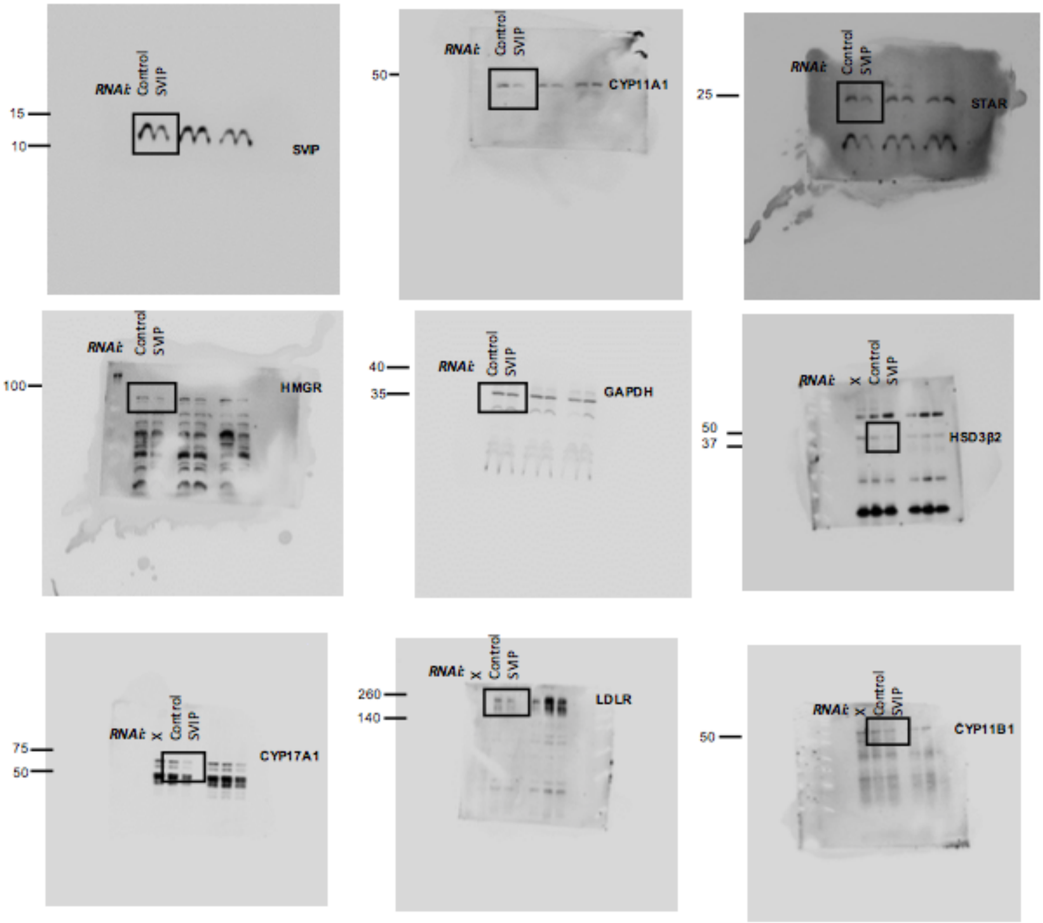
RNAi: Control SVIP
15
10
SVIP
50
CYP11A1
25
STAR
100
HMGR
40
35
GAPDH
RNAi: X Control SVIP
50
37
HSD3β2
75
50
CYP17A1
260
140
LDLR
50
CYP11B1

N

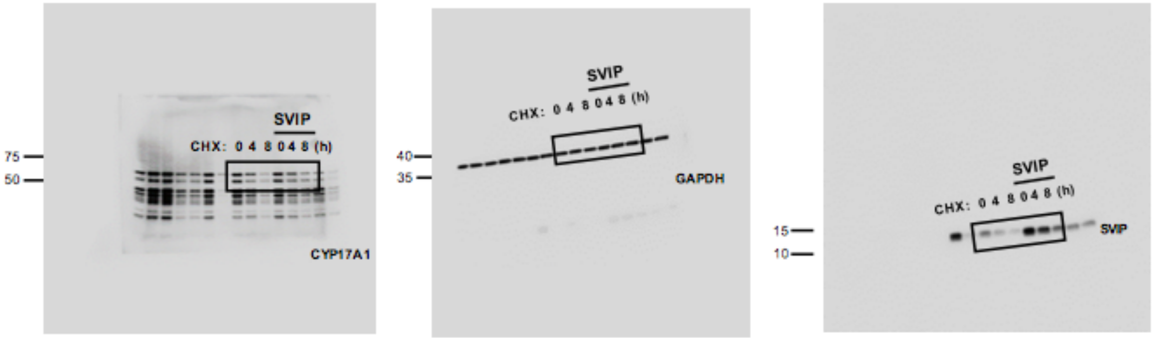
SVIP
CHX: 0 4 8 0 4 8 (h)
75
50
CYP17A1
40
35
GAPDH
15
10
SVIP

O

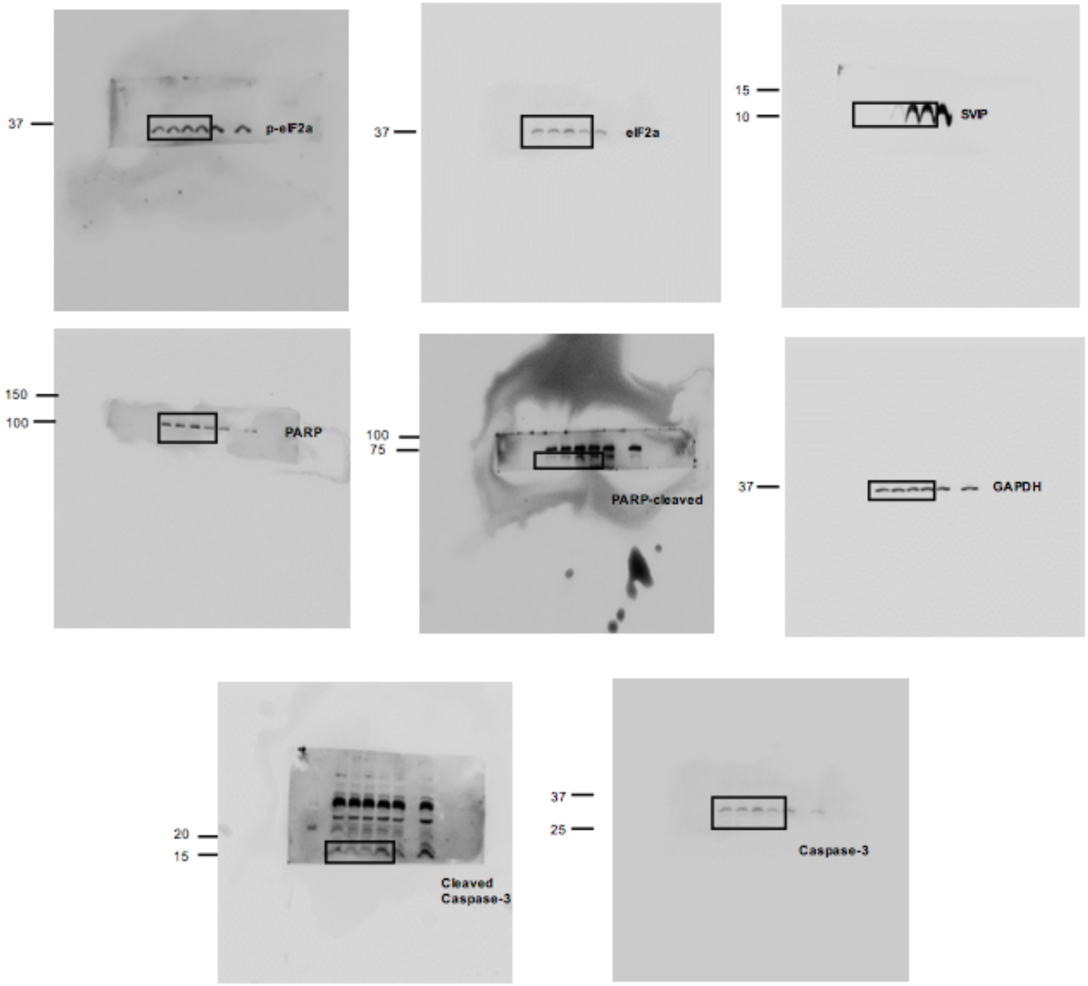
37
p-eIF2a
37
eIF2a
15
10
SVIP
150
100
PARP
100
75
PARP-cleaved
37
GAPDH
20
15
Cleaved
Caspase-3
37
25
Caspase-3

P

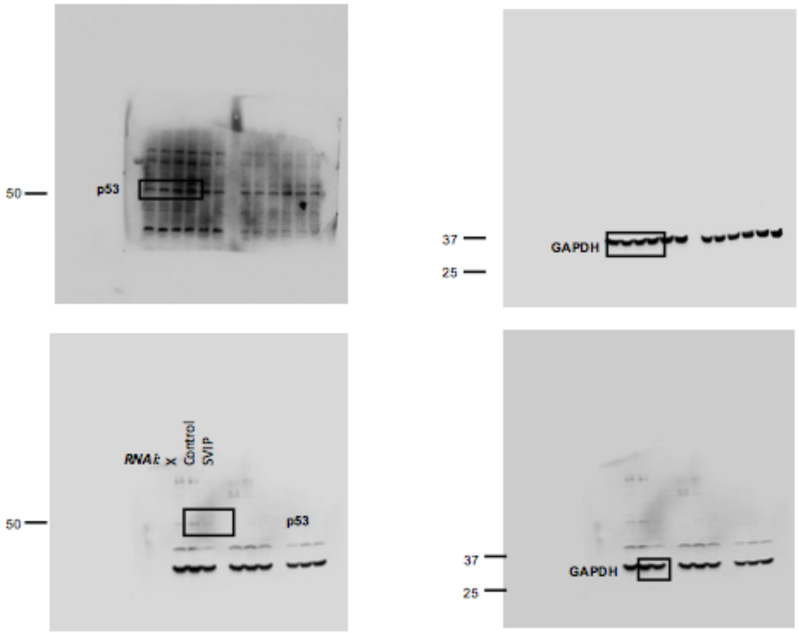
50
p53
37
25
GAPDH
RNAi:
Control
SVIP
50
p53
37
25
GAPDH

R

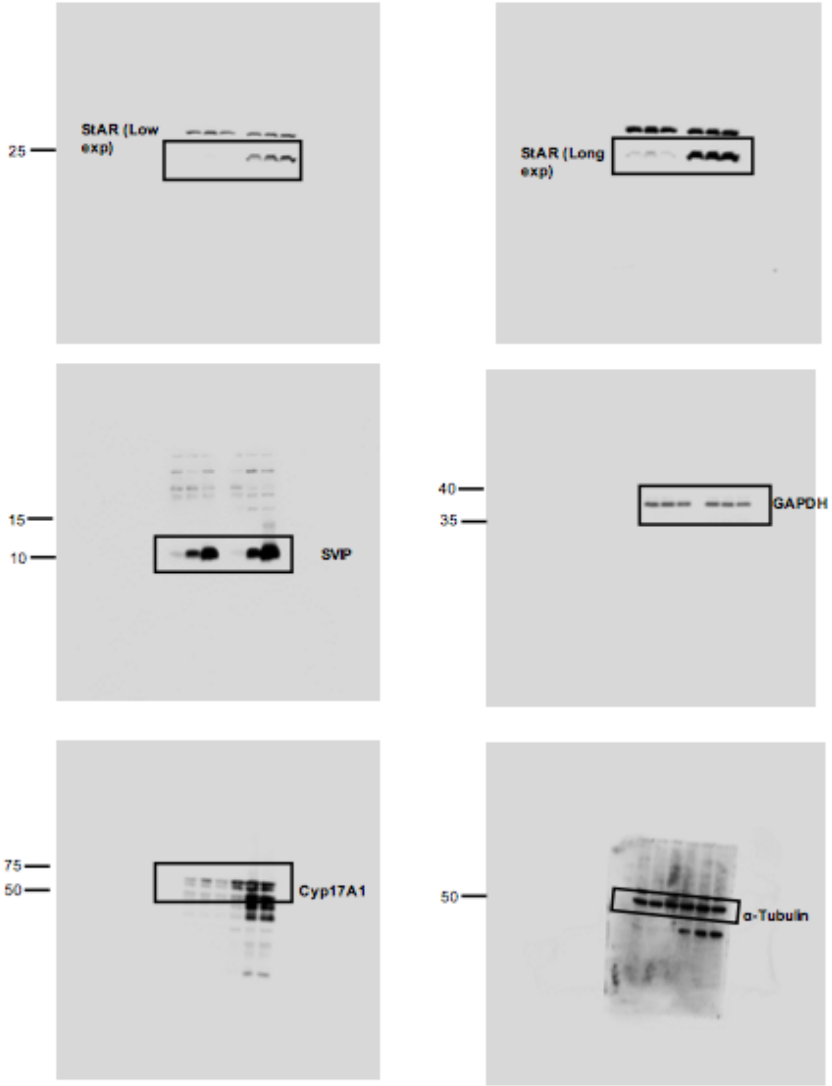

Supplementary Figure 5. Display of original blots. Images for **(A)** Figure 1A, **(B)** Figure 1B, **(C)** Figure 1C, **(D)** Figure 5A, **(E)** Figure 5B, **(F)** Figure 5C, **(G)** Figure 5D, **(H)** Figure 5F, **(I)** Figure 5G, **(J)** Figure 6A, **(K)** Figure 6B, **(L)** Figure 7B, **(M)** Figure 7C, **(N)** Figure 7D, **(O)** Figure 8A-membranes were cut prior to hybridization with antibodies, **(P)** Figure 8C, and **(R)** Figure 8C.
